# Supplementary material for: Combinatorial multimer staining and spectral flow cytometry facilitate quantification and characterization of polysaccharide-specific B cell immunity
Source: Commun Biol. 2023 Oct 28;6:1095. doi: 10.1038/s42003-023-05444-3 (PMC10613281; doi:10.1038/s42003-023-05444-3)
Supplement: Supplementary file 2 — Supplementary Figures and Tables [file 42003_2023_5444_MOESM2_ESM.pdf]

Supplementary Figures and Tables for:

Combinatorial multimer staining and spectral flow cytometry facilitate quantification and characterization of polysaccharide-specific B cell immunity

Dennis Hoving, Alexandre H.C. Marques, Wesley Huisman, Beckley A. Nosoh, Alicia C. de Kroon, Oscar R.J. van Hengel, Bing-Ru Wu, Rosanne A.M. Steenbergen, Pauline M. van Helden, Britta C. Urban, Nisha Dhar, Daniela M. Ferreira, Gaurav Kwatra, Cornelis H. Hokke and Simon P. Jochems

This file contains in order:

Supplementary Fig. 1

Supplementary Fig. 2

Supplementary Fig. 3

Supplementary Fig. 4

Supplementary Fig. 5

Supplementary Fig. 6

Supplementary Table 1

Supplementary Table 2

Supplementary Table 3

Supplementary Table 4

Supplementary Table 5

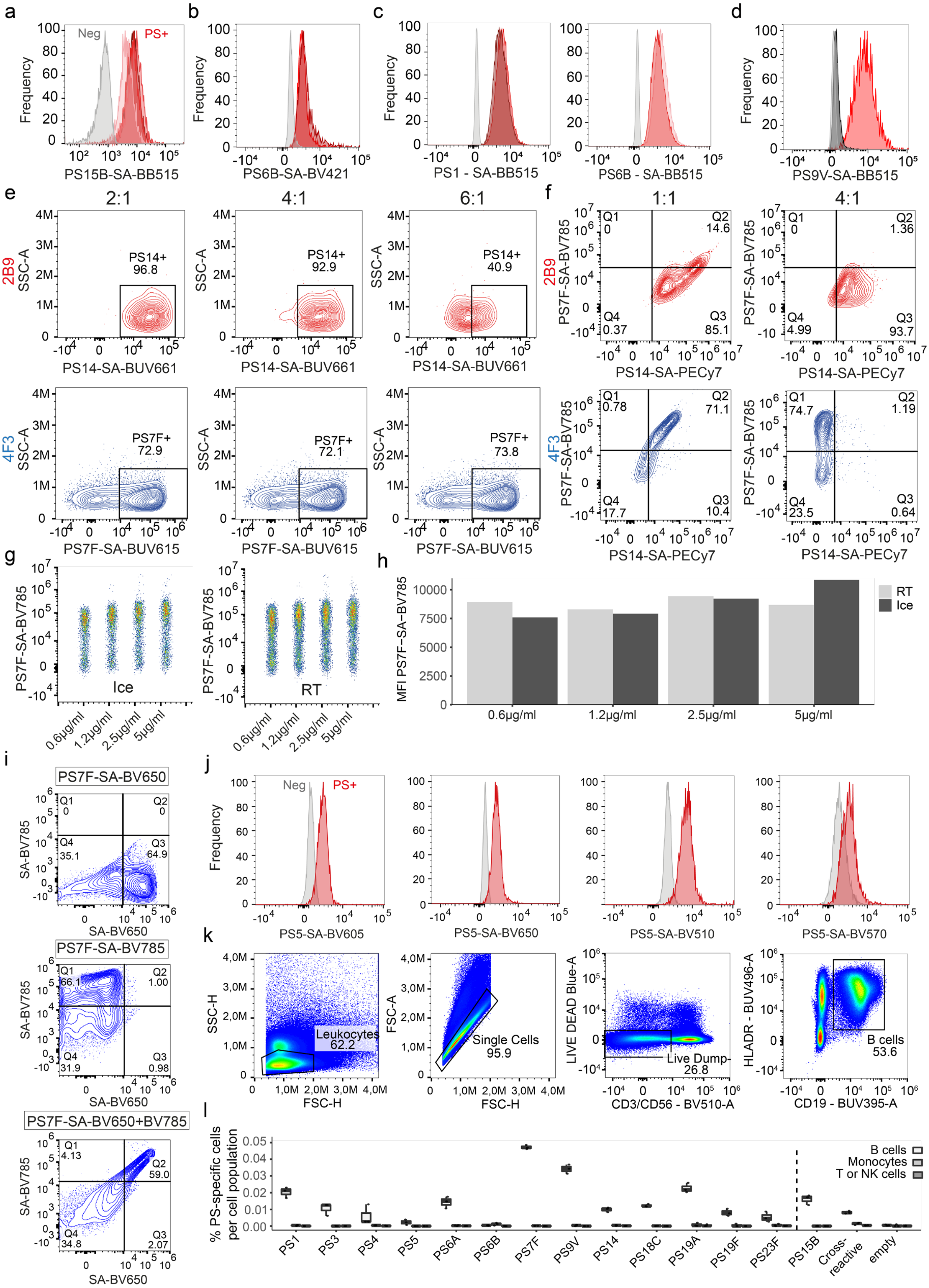
a
Neg
PS+
Frequency
PS15B-SA-BB515
b
PS6B-SA-BV421
c
PS1 - SA-BB515
PS6B - SA-BB515
d
PS9V-SA-BB515
e
2:1
4:1
6:1
2B9
SSC-A
PS14+
96.8
92.9
40.9
PS14-SA-BUV661
4F3
PS7F+
72.9
72.1
73.8
PS7F-SA-BUV615
f
1:1
4:1
PS7F-SA-BV785
Q1
0
Q2
14.6
1.36
Q4
0.37
4.99
Q3
85.1
93.7
Q1
0.78
74.7
Q2
71.1
1.19
Q4
17.7
23.5
Q3
10.4
0.64
PS14-SA-PECy7
g
Ice
RT
0.6µg/ml
1.2µg/ml
2.5µg/ml
5µg/ml
h
MFI PS7F−SA−BV785
RT
Ice
i
PS7F-SA-BV650
Q1 0
Q2 0
Q4 35.1
Q3 64.9
SA-BV785
SA-BV650
PS7F-SA-BV785
Q1 66.1
Q2 1.00
Q4 31.9
Q3 0.98
PS7F-SA-BV650+BV785
Q1 4.13
Q2 59.0
Q4 34.8
Q3 2.07
j
PS5-SA-BV605
PS5-SA-BV650
PS5-SA-BV510
PS5-SA-BV570
k
SSC-H
FSC-H
Leukocytes
62.2
FSC-A
Single Cells
95.9
LIVE DEAD Blue-A
CD3/CD56 - BV510-A
Live Dump-
26.8
HLADR - BUV496-A
CD19 - BUV395-A
B cells
53.6
l
% PS-specific cells
per cell population
B cells
Monocytes
T or NK cells
PS1
PS3
PS4
PS5
PS6A
PS6B
PS7F
PS9V
PS14
PS18C
PS19A
PS19F
PS23F
PS15B
Cross-
reactive
empty

Supplementary Fig. 1. Optimization and validation of assay conditions. a, Histogram of four batches of PS15B-SA-BB515 multimers (PS+; shades of red) prepared on different 4 days and recorded in 2 different readings, showing similar signal intensity, normalised to mode. Control unstained beads are depicted in grey (Neg). **b**, Signal of PS6B-SA-BV421 on specific antisera-covered compensation beads, normalised to mode, using multimers made from the same batch of biotinylated PS6B, either kept in at 4ºC (dark red) or -80ºC (light red) and multimerized with SA-BV421 three months later. **c**, Histograms of PS-SA-BB515 multimers, using freshly biotinylated (light red) PS1 (left panel) and PS6B (right panel) or biotinylated PS stored at -80ºC for 8-12 months (dark red), normalised to mode. Histograms include negative beads (grey). **d**, Specificity of antisera shown by signal of PS9V-SA-BB515 on compensation beads coupled to autologous antiserum pool R (red), heterologous antiserum factor 6C (black), or without antiserum (grey), normalised to mode. **e**, Spectral flow cytometry density plots showing 2B9 (red; PS14-specific) and 4F3 (blue; PS7F-specific) clones binding PS7F-SA-BV785 or PS14-SA-PECy7 multimers prepared with a PS:SA ratio of 2:1, 4:1 or 6:1, respectively. **f**, Spectral flow cytometry results of clone 2B9 (red; PS14-specific) and 4F3 (blue; PS7F-specific) stained simultaneously with PS7F-SA-BV785 and PS14-SA-PECy7 multimers, prepared with a PS:SA ratio of 1:1 or 4:1. The top right quadrant indicates double positive cells, which points to non-specific binding. **g**, Density plots of 4F3 (PS7F-specific) clones stained with various concentrations of PS7F-SA-BV785 multimers on ice for 30 minutes or at room temperature (RT) for 15 minutes. **h**, Bar plots showing the multimer MFI signal from PBMCs of a donor with no known PCV13 vaccination, stained with various concentrations of PS7F-SA-BV785 multimers on ice (30 minutes) or at room temperature (RT; 15 minutes). **i**, Spectral flow cytometry density plots showing 4F3 (PS7F-specific) clones stained with PS7F-SA-BV650 (top panel), PS7F-SA-BV785 (middle panel) or both simultaneously (bottom panel). **j**, Signal of discarded fluorochromes. Biotinylated PS5 in multimer with SA conjugated to different fluorochromes were bound to compensation beads using PS-specific antiserum (PS+; red), normalised to mode. Negative beads are shown for control (Neg;

grey). **k**, Gating strategy of PBMCs stained with the panel indicated in (Supplementary table 1). **l**, Frequencies of PS-specific cells in B cells ($SSC^{low}CD3^{-}CD56^{-}CD19^{+}HLADR^{+}$, light grey), monocytes ($SSC^{high}CD3^{-}CD56^{-}CD19^{-}$, darker grey) or T or NK cells ($SSC^{low}CD3^{+}CD56^{+}CD19^{-}$, darkest shade of grey) in PBMCs from a donor 3 weeks post-PCV13 vaccination.

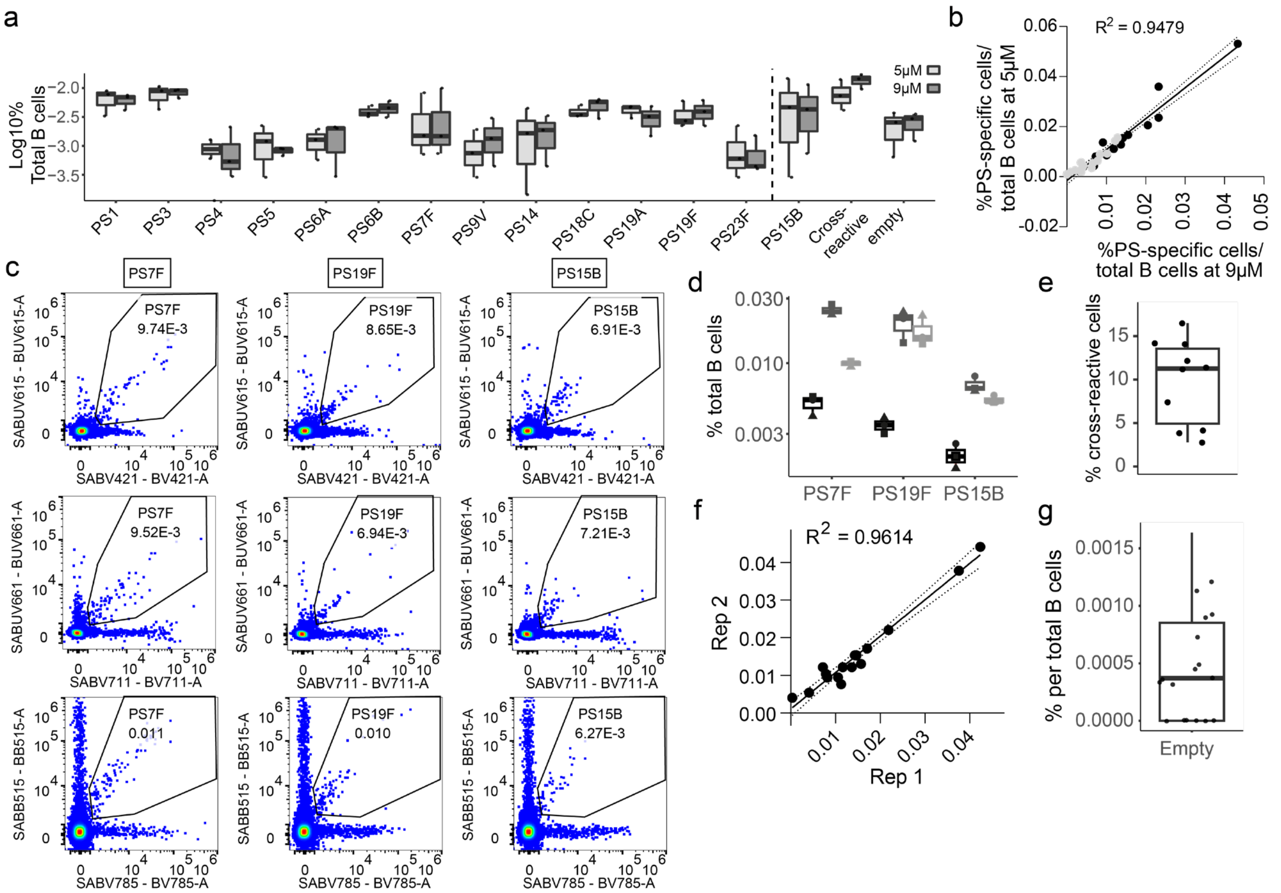

Supplementary Fig. 2. Validation of PBMCs probing with PS-SA multimers. a, Frequency of PS-specific cells in PBMCs from 3 donors with assumed no PCV13 vaccination, stained with 5µM (light grey) or 9µM (dark grey) PS-SA multimers for 14 serotypes. **b**, Correlation plot showing similarity between the staining of PBMCs with different concentrations in (a). Statistical analysis by Pearson correlation. **c-d**, example flow cytometry density plots (c) and frequencies (d) of PS-specific cells. Three donors (scales of grey) were stained each with three different SA-fluorochrome combinations (circle BV421/BUV615, triangle BV711/BUV661 and square BV785/BB515) for PS7F, PS19F and PS15B. Cells were pre-gated on live singlet $SSC^{low}CD7^{-}CD19^{+}$ cells. **e**, Percentage of cross-reactive cells among all PS-positive in all donors from Fig. 3e. **f**, Correlation between two experimental duplicates of stained PBMCs from one donor 3 weeks post-PCV13. The black lines indicates linear regression (R^2=0.9614) and the 95% confidence interval. Statistical analysis by Pearson correlation. **g**, Frequency of cells in the “empty” category ($BUV615^{+}BV711^{+}$) from all donors from (Fig. 3) to

indicate the background and lower limit of detection. Average, the standard deviation and individual results are shown.

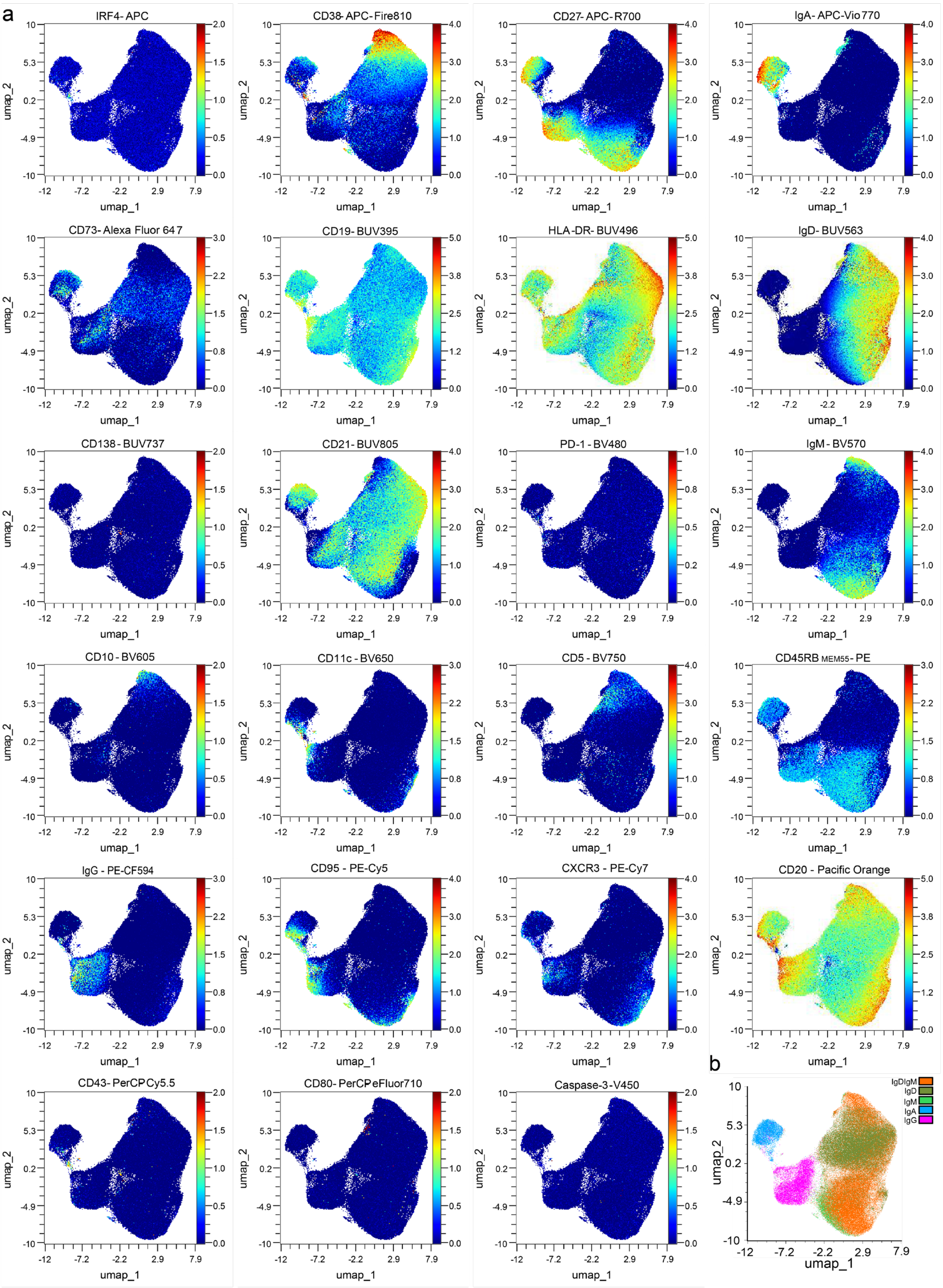
a
IRF4- APC
CD38- APC- Fire810
CD27- APC- R700
IgA- APC-Vio770
CD73- Alexa Fluor 647
CD19- BUV395
HLA-DR- BUV496
IgD- BUV563
CD138 - BUV737
CD21- BUV805
PD-1 - BV480
IgM - BV570
CD10 - BV605
CD11c - BV650
CD5 - BV750
CD45RB MEM55- PE
IgG - PE-CF594
CD95 - PE-Cy5
CXCR3 - PE-Cy7
CD20 - Pacific Orange
CD43- PerCPCy5.5
CD80- PerCPeFluor710
Caspase-3-V450
umap_1
umap_2
b
IgDIgM
IgD
IgM
IgA
IgG

Supplementary Fig. 3. Marker expression distribution across the UMAP. a, Density plots from all clustered B cells from (Fig. 4). **b**, Clusters of IgD^+IgM^+ (orange), IgD^+ (dark green), IgM^+ (light green), IgA^+ (blue) and IgG^+ (magenta) gated B cells on a UMAP, from (Fig. 4a-b).

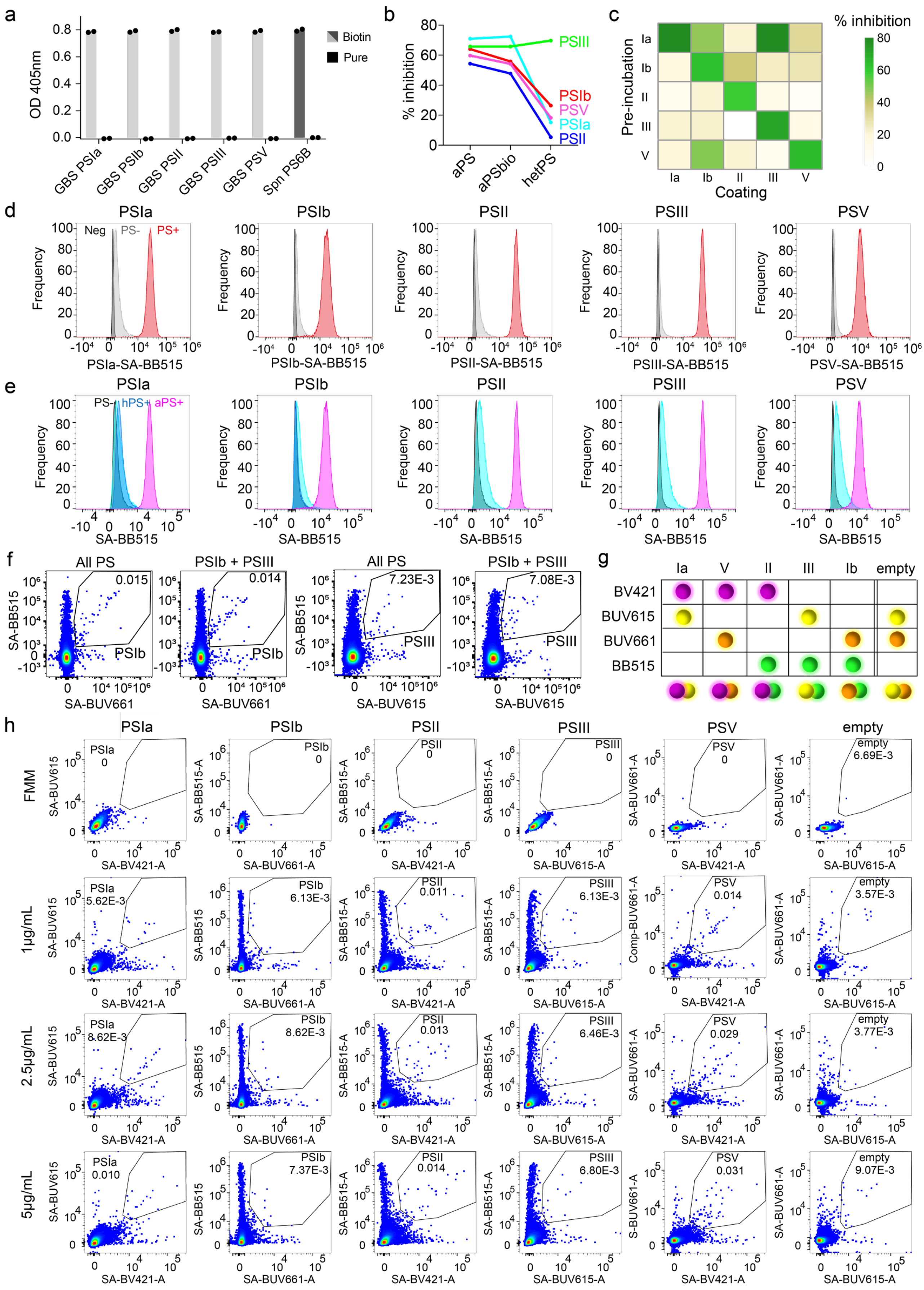
a
b
c
d
e
f
g
h
OD 405nm
Biotin
Pure
GBS PSIa
GBS PSIb
GBS PSII
GBS PSIII
GBS PSV
Spn PS6B
% inhibition
aPS
aPSbio
hetPS
PSIII
PSIb
PSV
PSIa
PSII
Pre-incubation
Coating
Frequency
Neg
PS-
PS+
PSIa-SA-BB515
PSIb-SA-BB515
PSII-SA-BB515
PSIII-SA-BB515
PSV-SA-BB515
hPS+
aPS+
SA-BB515
All PS
PSIb + PSIII
SA-BUV661
SA-BUV615
BV421
BUV615
BUV661
BB515
empty
FMM
1µg/mL
2.5µg/mL
5µg/mL
SA-BV421-A
SA-BUV661-A
SA-BUV615-A
SA-BB515-A
SA-BUV661-A

Supplementary Fig. 4. Optimization of GBS PS biotinylation and PS-multimer formation. a, Bar plots with biotin ELISA results showing the efficacy of biotin incorporation in CDAP-activated GBS PS (light grey) and pneumococcal PS6B as positive control (dark grey). Untreated PS are shown as negative control (black). Blank-corrected OD values are shown. For each PS, levels were measured in duplicate, indicated by symbols with average depicted by bars. **b**, Competition ELISA showing the percentage of antibody blocking by biotinylated and non-modified PS. For each PS, inhibition of detection by pre-incubation of pooled donor samples (confirmed presence of serotype-specific antibodies), with autologous unmodified PS (aPS), autologous biotinylated PS (aPSbio), heterologous unmodified PS (hetPS) is shown. Inhibition indicates the reduction in OD signal in pre-absorbed versus non-pre-absorbed serum. Average of duplicates is shown. **c**, Competition ELISA as (b), showing all autologous and heterologous combinations of non-biotinylated PS, to show cross-reactivity of serotype-specific antibodies. Average of duplicates is shown. **d**, Histograms showing fluorescent signal of PS-SA multimers on compensation beads alone (Neg; black), with PS-SA-multimers (PS-; grey) or beads coupled with PS-specific autologous antiserum and stained with PS-SA-multimers (PS+; red), normalised to mode. **e**, Histograms showing signal of compensation beads with PS-SA-multimers alone without antiserum to indicate background (PS-; black) or compensation beads coupled to autologous serotype-specific antiserum and stained with autologous (aPS+; magenta) or heterologous (hPS+; light blue are combined heterologous PS, dark blue are heterologous PSIa/PSIb) PS-SA-multimers, normalised to mode. **f**, Frequency of PSIb and PSIII-specific cells in pooled PBMCs from 6 South African donors, after staining for all 5 serotypes (PSIa, PSIb, PSII, PSIII and PSV) or solely 2 (PSIb and PSIII). **g**, Barcode method of combinatorial staining patterns for GBS PS-SA multimers. The four fluorochromes are indicated in rows and the various serotypes in columns, including an empty colour combination for background control. **h**, Staining of pooled PBMCs of South African donors, with viability dye, CD3, CD19 and 3 concentrations of PS-SA-multimers: 1µg/mL,

2.5µg/mL and 5µg/mL. The fluorescence minus multimer (FMM) control sample was made by pooling all samples and staining with everything but multimers.

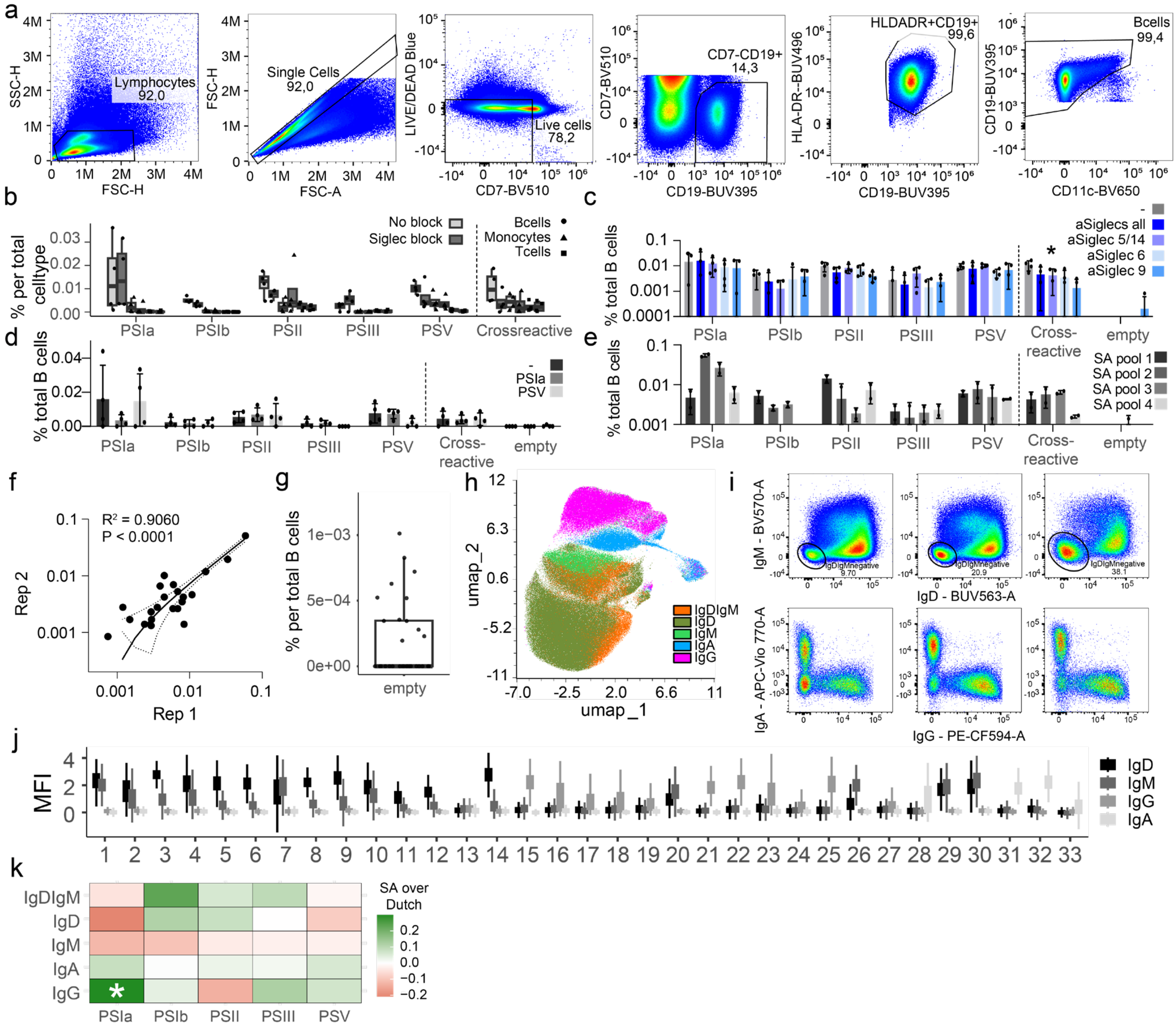

Supplementary Fig. 5. Verification on GBS PS-multimer staining. a, Gating strategy for Dutch and South African PBMCs stained with the panel in (Supplementary table 4). **b**, Frequencies of PS-specific cells in B cells ($SSC^{low}CD3^{-}CD19^{+}HLADR^{+}$, circles), monocytes ($SSC^{high}CD3^{-}CD19^{-}$, triangles) and T cells ($SSC^{low}CD3^{+}CD19^{-}$, squares), in pooled PBMCs from South African donors. Siglec receptors were either blocked (dark grey) with 10µg/mL each of anti-Siglec-5/14 (RnD systems, MAB10721-SP), anti-Siglec-6 (RnD systems, MAB2859-SP), anti-Siglec-9 (RnD systems, MAB1139-100), or non-blocked (light grey). Average and standard deviation are shown. **c**, Signal of pooled PBMCs of South African donors pre-blocked (shades of blue) with 10µg/mL anti-Siglec-5/14 (RnD systems, MAB10721-SP), anti-Siglec-6 (RnD systems, MAB2859-SP), anti-Siglec-9 (RnD systems,

MAB1139-100), 30µg/mL anti-Siglec all combined (10µg/mL each) or nothing (grey), and stained with viability dye, CD3, CD19 and PS-SA multimers. **d**, Frequency of PS-specific B cells in pooled PBMCs from South African donors, pre-blocked with 10µg/mL each anti-Siglec-5/14, anti-Siglec-6 and anti-Siglec-9, in combination with nothing (black) or 100µg/mL PSIa (dark grey) or PSV (light grey). **e**, Frequencies of serotype-specific B cells in 4 different South African combined donor PBMC pools (shades of grey). Average, standard deviation and individual measurements from two technical duplicates are shown. **f**. Pearson correlation plot showing the similarity between the technical duplicates from (e). **g**, Frequency of cells in the "empty" category (BUV615^{+}BUV661^{+}) from all donors from (Fig. 5) to indicate the background and lower limit of detection. **h**, Clusters of IgD^{+}IgM^{+} (orange), IgD^{+} (dark green), IgM^{+} (light green), IgA^{+} (blue) and IgG^{+} (magenta) B cells on a UMAP. **i**, Example flow cytometry density plots from 3 donors from Figure 5, showing signal intensity of IgD and IgM (top panels) on pre-gated B cells (a) and IgG and IgA (bottom panels) on IgDIgM-negative cells. **j**, Arcsine transformed mean fluorescence intensity (MFI) of IgD, IgM, IgG and IgA-isotypes in each cluster from Fig. 5e-f. **k**, Heatmap showing the difference in confidence intervals for each serotype and isotype between Dutch and South African (SA) donors, assessed through a two-tailed test based on a linear regression model with wild bootstrap simulation at 9999 resamples. Bonferroni-Hochberg correction for multiple testing was employed. Stars indicate which PS-specific B cells and isotype are significantly associated to South African donors. (* $p<0.05$)

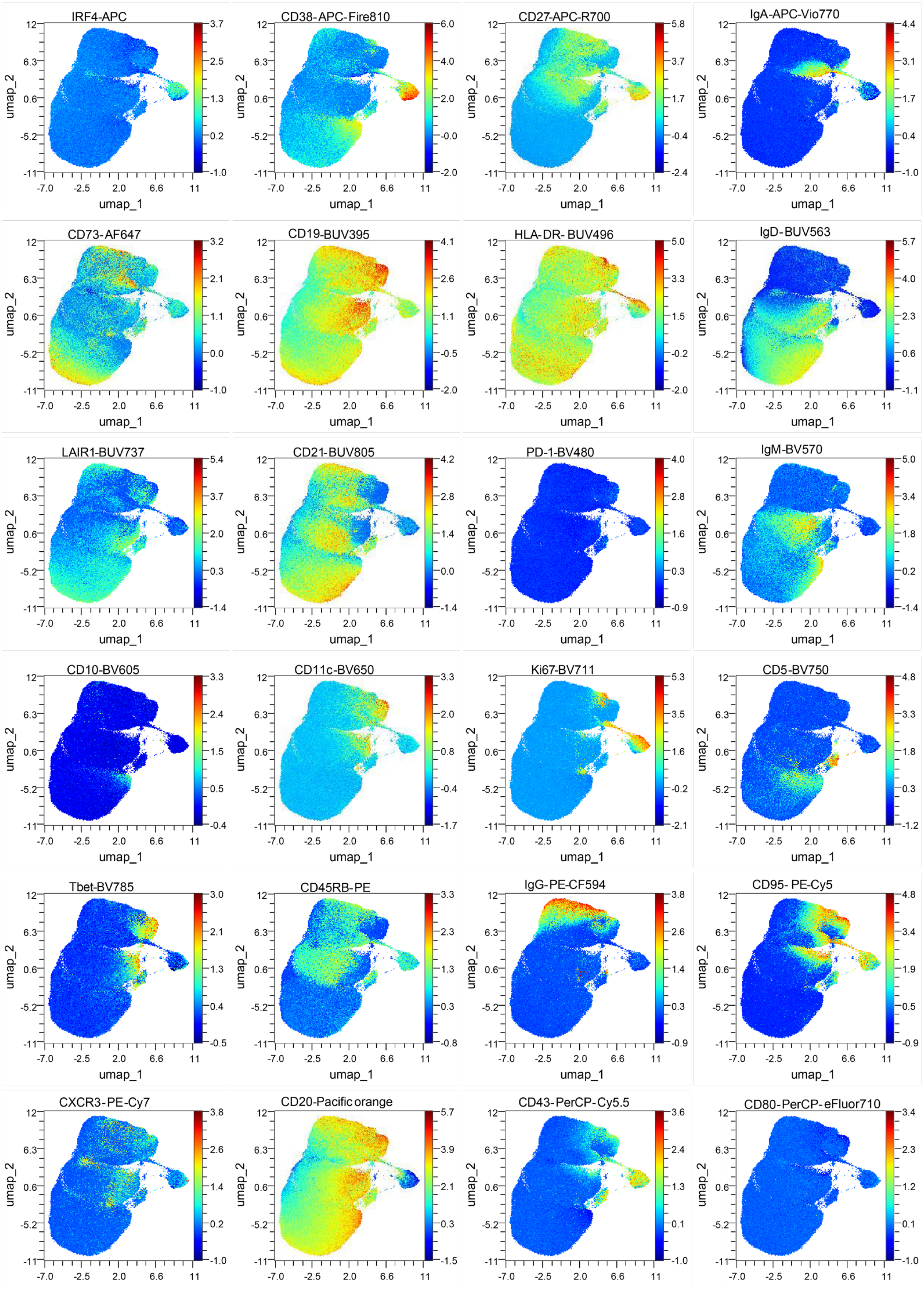
IRF4-APC
CD38-APC-Fire810
CD27-APC-R700
IgA-APC-Vio770
CD73-AF647
CD19-BUV395
HLA-DR- BUV496
IgD-BUV563
LAIR1-BUV737
CD21-BUV805
PD-1-BV480
IgM-BV570
CD10-BV605
CD11c-BV650
Ki67-BV711
CD5-BV750
Tbet-BV785
CD45RB-PE
IgG-PE-CF594
CD95- PE-Cy5
CXCR3-PE-Cy7
CD20-Pacific orange
CD43-PerCP-Cy5.5
CD80-PerCP- eFluor710
umap_1
umap_2

Supplementary Fig. 6. Marker expression distribution across the UMAP of GBS samples. Density plots from all clustered B cells from (Fig. 5).

Supplementary table 1							
**Marker/dye**	**Fluorochrome**	**Clone**	**Isotype**	**Company**	**Catalogue**	**Dilution**	**Surface/intracel.**
CD3	BV510	OKT3	Mouse IgG2a. κ	Biolegend	317332	100	Surface
CD56	BV510	HCD56	Mouse IgG1. κ	Biolegend	318340	50	Surface
CD19	BUV395	HIB19	Mouse IgG1. κ	BD	740287	200	Surface
HLA-DR	BUV496	G46-6	Mouse IgG2a. κ	BD	749866	200	Surface
IgD	BUV563	I-A6-2	Mouse IgG2a. κ	BD	741394	400	Surface
IgM	BV570	MHM-88	Mouse IgG1. κ	Biolegend	314517	400	Surface
IgA	APC-Vio770	IS11-8E10	Mouse IgG1κ	Miltenyi	130-113-999	1600	Surface
IgG	PE-CF594	G18-145	Mouse IgG1. κ	BD	562538	400	Surface
CD138	BUV737	MI15	Mouse IgG1. κ	BD	612834	50	Surface
CD38	APC-Fire810	HIT2	Mouse IgG1. κ	Biolegend	303550	100	Surface
CD10	BV605	HI 10 a	Mouse IgG1. κ	Biolegend	312222	100	Surface
CD11c	BV650	Bu15	Mouse IgG1. κ	Biolegend	337237	400	Surface
CD20	Pacific orange	HI47	Mouse IgG3	ThermoFisher	MHCD2030	20	Surface
CD21	BUV805	BLy4	Mouse IgG1. κ	BD	742008	200	Surface
CD27	APC-R700	M-T271	Mouse IgG1. κ	BD	565116	100	Surface
CD43	PerCP-Cy5.5	1G10	Mouse IgG1. κ	BD	563521	400	Surface
$CD45RB_{MEM55}$	PE	MEM-55	Mouse IgG2b. κ	Biolegend	310204	400	Surface
CD5	BV750	L17F12	Mouse IgG2a. κ	BD	747090	200	Surface
CD73	AF647	AD2	Mouse IgG1	Abcam	243083	200	Surface
CD95	PE-Cy5	DX2	Mouse IgG1. κ	Biolegend	305610	400	Surface
CD80	PerCP-eFluor710	16-10A1	Hamster IgG	ThermoFisher	46-0801-82	200	Surface
CXCR3	PE-Cy7	G025H7	Mouse IgG1. κ	Biolegend	353719	1200	Surface
PD-1	BV480	EH12.1	Mouse IgG1. κ	BD	566112	100	Surface
IRF4	APC	REA201	Human IgG1	Miltenyi	130-100-915	200	Intracellular
Caspase-3	V450	C92-605	Rabbit IgG	BD	560627	100	Intracellular
SA-BB515	BB515	NA	NA	BD	564453	NA	NA
SA-BUV615	BUV615	NA	NA	BD	613013	NA	NA
SA-BUV661	BUV661	NA	NA	BD	612979	NA	NA
SA-BV421	BV421	NA	NA	BD	563259	NA	NA
SA-BV711	BV711	NA	NA	Biolegend	405241	NA	NA
SA-BV785	BV785	NA	NA	Biolegend	405249	NA	NA
Live/Dead	Blue	NA	NA	ThermoFisher	L34962	500	NA

Supplementary table 1. Antibody list for Spn analysis.

Supplementary table 2							
cluster	timepoint	obs_estimate	unbiased_estimate	ci_limit_0.05	ci.adj_limit_0.025	signif_ci	signif_ci.adj
elbow07	M4	0.0387190677018709	0.0388413938599702	0.018539172120877	0.0146486905440044	1	1
elbow11	M4	0.0454526765194477	0.0454197192337327	0.0187444485062122	0.0132632268303365	1	1
elbow03	M4	0.095696755686353	0.0960637957209242	0.0520957451537589	0.0445783096409878	1	1
elbow09	M4	0.0125221006471866	0.0124811661635298	0.00513192131803552	0.00389584391165417	1	1
elbow13	M4	0.00615223040135064	0.00613942635972125	0.00253597562536561	0.00184346800988204	1	1
elbow22	M4	0.0201158966958282	0.0201486626538739	0.00809270135571964	0.00603222312614188	1	1
elbow05	M4	0.00907469424297034	0.00906400285652889	-0.000373093228988612	-0.00225446084750009	0	0
elbow06	M4	0.0180891120160918	0.0181043629341241	-0.000036215036735241[illegible]	-0.0034584209879452	0	0
elbow01	M4	-0.0024981382599618	-0.0024132966033086	-0.0132151651199272	-0.0152337353058856	0	0
elbow02	M4	0.0130494227051925	0.0131843989380414	-0.0097832443004755	-0.0142626183174471	0	0
elbow04	M4	0.00933073584338708	0.00938929388130626	-0.0416823581153119	-0.0513514973457047	0	0
elbow08	M4	-0.00513281401185176	-0.0051082719673856	-0.019118891888884	-0.021894343648739	0	0
elbow10	M4	-0.0231473980815505	-0.022952299350188	-0.0431189156675798	-0.0469479950871325	0	0
elbow12	M4	0.00644912123054663	0.00654913946661539	-0.00255756237309722	-0.00412868342618228	0	0
elbow14	M4	-0.00761641811329294	-0.00765142033868941	-0.0160808366775275	-0.0178036956686031	0	0
elbow15	M4	0.00355389260749793	0.00358463673558799	-0.000961425652625518	-0.00186405755249227	0	0
elbow16	M4	0.0132181150025836	0.0133113038246177	-0.00207482636801922	-0.00495417305237344	0	0
elbow17	M4	-0.00474035768153415	-0.00469576377506566	-0.011455676010764	-0.012707994428801	0	0
elbow18	M4	0.000183150183150183	0.00020787500969714	-0.00686073902871814	-0.00811112572691376	0	0
elbow19	M4	-0.00264732205181388	-0.00265344592169131	-0.00760313084755765	-0.00856506241830524	0	0
elbow20	M4	-0.00624627664103402	-0.00633196374608449	-0.0156518562839745	-0.0173514161278738	0	0
elbow21	M4	-0.020435225185977	-0.0205753313048421	-0.038636419949816	-0.0418786441611714	0	0
elbow23	M4	-0.00160144953991246	-0.00157102123058828	-0.00598616834210475	-0.00681272210889326	0	0
elbow24	M4	-0.00653317402456673	-0.00653876520719599	-0.0147338873531064	-0.0163171306459282	0	0
elbow25	M4	-0.00811143818790515	-0.00802723956142462	-0.0232008980978041	-0.025933154315713	0	0
elbow26	M4	-0.0207794000416989	-0.0206931481316721	-0.0415914211065924	-0.0456373908397772	0	0
elbow27	M4	0.000616279268627808	0.000638946994774594	-0.00219779376716285	-0.0027438291857353	0	0
elbow28	M4	-0.00439108075221043	-0.00442172252036754	-0.0102328711588892	-0.0112743278210812	0	0
elbow29	M4	-0.003956830616703	-0.0039928514185696	-0.00977113592871606	-0.0108868166055653	0	0
elbow30	M4	-0.0126474625316218	-0.0128184683128676	-0.0301570108575694	-0.0335984959939659	0	0
elbow31	M4	0.00681544093296104	0.00683167417119726	-0.0108668491794457	-0.0141446526499419	0	0
elbow32	M4	-0.00771034257342575	-0.00771362103385678	-0.0130500235349328	-0.0140847556191554	0	0
elbow33	M4	-0.00891705485092482	-0.00890945486128366	-0.0144818411428929	-0.0154814990882119	0	0
elbow34	M4	-0.00612131899947026	-0.00615487141352728	-0.0159743002418259	-0.017846783846472	0	0
elbow35	M4	-0.0221788159132166	-0.0221201276911835	-0.0353166393577675	-0.037693740775137	0	0

Supplementary table 2. Statistical results from linear regression model bootstrapping for Spn cross-sectional phenotype and vaccination analysis. The observed (obs_estimate) and estimated (unbiased_estimate) difference in proportion of clusters among total B cells comparing PCV13-vaccinated (M4) to non-vaccinated individuals were generated with an upper-sided linear regression model with wild bootstrap simulation (9999 resamples). Confidence intervals (ci) and Bonferroni's method adjusted ci (ci.adj) are shown, as well as statistical significance (signif_ci/ signif_ci.adj = 1).

Supplementary table 3							
**Marker/dye**	**Fluorochrome**	**Clone**	**Isotype**	**Company**	**Catalogue**	**Dilution**	**Surface/intracel.**
CD7	BV510	M-T701	Mouse IgG1. κ	BD	563650	100	Surface
CD19	BUV395	HIB19	Mouse IgG1. κ	BD	740287	200	Surface
HLA-DR	BUV496	G46-6	Mouse IgG2a. κ	BD	749866	200	Surface
IgD	BUV563	I-A6-2	Mouse IgG2a. κ	BD	741394	400	Surface
IgM	BV570	MHM-88	Mouse IgG1. κ	Biolegend	314517	200	Surface
IgA	APC-Vio770	IS11-8E10	Mouse IgG1κ	Miltenyi	130-113-999	1600	Surface
IgG	PE-CF594	G18-145	Mouse IgG1. κ	BD	562538	400	Surface
CD38	APC-Fire810	HIT2	Mouse IgG1. κ	Biolegend	303550	100	Surface
CD10	BV605	HI 10 a	Mouse IgG1. κ	Biolegend	312222	100	Surface
CD11c	BV650	Bu15	Mouse IgG1. κ	Biolegend	337237	400	Surface
CD20	Pacific orange	HI47	Mouse IgG3	ThermoFisher	MHCD2030	20	Surface
CD21	BUV805	BLy4	Mouse IgG1. κ	BD	742008	200	Surface
CD27	APC-R700	M-T271	Mouse IgG1. κ	BD	565116	100	Surface
CD43	PerCP-Cy5.5	1G10	Mouse IgG1. κ	BD	563521	400	Surface
$CD45RB_{MEM55}$	PE	MEM-55	Mouse IgG2b. κ	Biolegend	310204	400	Surface
CD5	BV750	L17F12	Mouse IgG2a. κ	BD	747090	200	Surface
CD73	AF647	AD2	Mouse IgG1	Abcam	243083	200	Surface
CD95	PE-Cy5	DX2	Mouse IgG1. κ	Biolegend	305610	400	Surface
CD80	PerCP-eFluor710	16-10A1	Hamster IgG	ThermoFisher	46-0801-82	400	Surface
CXCR3	PE-Cy7	G025H7	Mouse IgG1. κ	Biolegend	353719	300	Surface
PD-1	BV480	EH12.1	Mouse IgG1. κ	BD	566112	100	Surface
LAIR1	BUV737	DX26 (RUO)	Mouse IgG1. κ	BD	749446	400	Surface
IRF4	APC	REA201	Human IgG1	Miltenyi	130-100-915	200	Intracellular
Ki-67	BV711	Ki67	Mouse IgG1. κ	Biolegend	350515	400	Intracellular
T-bet	BV785	4B10	Mouse IgG1. κ	Biolegend	644835	100	Intracellular
SA-BB515	BB515	NA	NA	BD	564453	NA	NA
SA-BUV615	BUV615	NA	NA	BD	613013	NA	NA
SA-BUV661	BUV661	NA	NA	BD	612979	NA	NA
SA-BV421	BV421	NA	NA	BD	563259	NA	NA
Live/Dead	Blue	NA	NA	ThermoFisher	L34962	500	NA

Supplementary table 3. Antibody list for GBS analysis.

Supplementary table 4									
cluster	type	obs_estimate	unbiased_estimate	ci_limit_0.025	ci_limit_0.975	ci.adj_limit_0.005	ci.adj_limit_0.995	signif_ci	signif_ci.adj
IgG	PSIa	0.297912206130363	0.299039856317133	0.14975417051434	0.439840962827398	0.105861315750359	0.484584723888382	1	1
IgG	PSIII	0.120990436331345	0.120348081226637	0.00512145527064722	0.236209174402328	-0.0307200461421479	0.272579090230043	1	0
IgA	PSIb	0.00374230270620651	0.0041038714582251	-0.0731874274227158	0.0784250252830294	-0.0981078742549699	0.10619671130899	0	0
IgA	PSII	0.0282544701118572	0.0280538287595615	-0.105233745733272	0.16115871866462	-0.145758466096581	0.20240877434359	0	0
IgA	PSIII	0.0174878782833328	0.0175517034327768	-0.0504831232030867	0.0837804102267365	-0.0723371707456517	0.105155227086669	0	0
IgA	PSV	0.0590732531357532	0.0589324807687921	-0.00488407139076333	0.12280419906185	-0.0271181827890558	0.142750081950293	0	0
IgA	PSIa	0.0825265914546965	0.0825261948947213	-0.0208315201777741	0.186392211228752	-0.0533861301148082	0.219659165429992	0	0
IgD	PSIb	0.114279492788851	0.114999559615973	-0.0254726331380426	0.252777487961871	-0.0714173612445071	0.298212952356342	0	0
IgD	PSII	0.0804357654532321	0.0803485784807978	-0.0343924637993539	0.192919831970397	-0.0668466782092957	0.228838275655617	0	0
IgD	PSIII	-0.0010630152675607	-0.00148774520505988	-0.242029634851683	0.241140763767503	-0.318355949156198	0.319648596447281	0	0
IgD	PSV	-0.086894529082029	-0.0863578701529576	-0.236582454662417	0.0608480849133081	-0.28465198825595	0.111418006077663	0	0
IgD	PSIa	-0.210516905278866	-0.208617418338795	-0.457612174854376	0.027181080633052	-0.544242098592279	0.104652171987272	0	0
IgDIgM	PSIb	0.230801328795981	0.231161286988667	-0.00438615519772776	0.462282258793965	-0.0765229108043725	0.544048137690967	0	0
IgDIgM	PSII	0.0603747901295082	0.0599010533399151	-0.0817671783205953	0.202582014900993	-0.126428528093155	0.246201703592698	0	0
IgDIgM	PSIII	0.0917518087972635	0.0949304976525217	-0.143318905752151	0.31734454092602	-0.209255963778439	0.389874649361361	0	0
IgDIgM	PSV	-0.0153449328449335	-0.0156603894889066	-0.227378714682933	0.19683138191507	-0.297455550715123	0.260827421255727	0	0
IgDIgM	PSIa	-0.0480003198516886	-0.0471670132007757	-0.234020395465675	0.136497218184659	-0.295560259870355	0.189966340780082	0	0
IgG	PSIb	0.0379203127532006	0.0376857804751357	-0.137550216409217	0.212923221276193	-0.195321775849025	0.266616318800949	0	0
IgG	PSII	-0.135219839626075	-0.135009395399472	-0.354144100924798	0.0809770097187282	-0.429366943269422	0.148928214196056	0	0
IgG	PSV	0.0683729464979467	0.067544559033488	-0.0664245684036486	0.202600617264328	-0.103455824488959	0.241166797812673	0	0
IgM	PSIb	-0.10265252795333	-0.102201983685883	-0.2969608707855	0.0898658093530763	-0.35449524937631	0.153306546594738	0	0
IgM	PSII	-0.0338451860685226	-0.0337354451266717	-0.136917589525561	0.067127647711779	-0.167314891572706	0.0979144678319177	0	0
IgM	PSIII	-0.0246216535989263	-0.0247651979896518	-0.13063977350382	0.0812224856260448	-0.162315552306208	0.114364334802359	0	0
IgM	PSV	-0.0252067377067377	-0.0255429139515515	-0.190976250204212	0.135259826247834	-0.242361959797995	0.184721834662163	0	0
IgM	PSIa	-0.121921572454504	-0.120939621350069	-0.310653979024049	0.0655352417517152	-0.379912991307615	0.126446504778936	0	0

Supplementary table 4. Statistical results from linear regression model bootstrapping for GBS isotype and population analysis. Table shows the observed (obs_estimate) and estimated (unbiased_estimate) difference in proportions of clusters among total B cells for a given serotype-specificity and B cell isotype, comparing South African- with Dutch donors. The analysis was performed using a two-sided linear regression model with wild bootstrap simulation (9999 resamples). Confidence intervals (ci) and Bonferroni's method adjusted ci (ci.adj) are shown, as well as statistical significance (signif_ci/ signif_ci.adj = 1).

Supplementary table 5									
cluster	type	obs_estimate	unbiased_estimate	ci_limit_0.025	ci_limit_0.975	ci.adj_limit_0.005	ci.adj_limit_0.995	signif_ci	signif_ci.adj
elbow06	PSIb	1,08E-03	1,08E-03	6,56E-04	1,50E-03	5,14E-04	1,63E-04	1	1
elbow14	PSII	0.0126875708961271	0.0127517387360139	0.00342290586185557	0.0216497293647453	0.0004951804991052	0.0249148379803903	1	1
elbow20	PSIa	0.039257444361733	0.0391506993020294	0.0106205177344011	0.0681754258113157	0.00146429143611112	0.0779807106691606	1	1
elbow31	PSIa	0.0601316268135356	0.0601352378480176	0.0220846436838107	0.0985433461820823	0.00878887268485867	0.11032963397321	1	1
elbow31	PSV	0.0666205757332066	0.0665026687064256	0.03271904742664	0.100336792367083	0.0225189647807544	0.111336940022469	1	1
elbow22	PSIa	0.066972619152292	0.0669317225693528	0.025005414689214	0.109269225564805	0.0130963451706423	0.122093372752177	1	1
elbow26	PSV	0.0774340381723222	0.077346458162919	0.0272364866545509	0.127828463131688	0.0104859658561076	0.142727641269059	1	1
elbow24	PSIa	0.126869289581038	0.127306786528142	0.0692927514343191	0.183538832522994	0.0508724214131485	0.201947525137912	1	1
elbow09	PSV	-1,80E-02	-1,80E-02	-3,05E-02	-5,55E-03	-3,47E-02	0	1	0
elbow05	PSIII	-3,47E-03	-3,46E-03	-1,18E-02	5,55E-03	-1,39E-02	8,33E-03	0	0
elbow12	PSV	-2,08E-03	-2,10E-03	-6,96E-03	2,92E-03	-8,41E-04	4,44E-06	0	0
elbow17	PSV	-1,04E-03	-1,02E-04	-5,20E-03	2,78E-03	-6,25E-03	4,16E-03	0	0
elbow11	PSV	-3,47E-04	-3,44E-04	-1,83E-03	1,13E-03	-2,27E-03	1,65E-03	0	0
elbow01	PSIb	-6,41E-07	-6,40E-05	-5,83E-04	4,49E-05	-7,43E-04	5,82E-04	0	0
elbow28	PSIb	0	-1,72E-06	-2,78E-03	2,78E-03	-2,78E-03	2,78E-03	0	0
elbow01	PSII	4,47E-04	4,56E-04	-2,04E-03	2,93E-03	-2,78E-04	3,85E-04	0	0
elbow11	PSIb	6,07E-04	6,10E-04	-1,81E-04	1,41E-03	-4,63E-04	1,79E-03	0	0
elbow17	PSIb	6,94E-04	7,04E-04	-2,08E-03	3,47E-03	-2,78E-03	4,17E-03	0	0
elbow17	PSIII	1,39E-03	1,43E-03	-2,78E-03	5,55E-03	-4,16E-03	6,94E-03	0	0
elbow01	PSIa	1,69E-03	1,70E-03	-2,15E-03	5,43E-03	-3,13E-03	6,60E-03	0	0
elbow06	PSII	0.00047846889952153	0.00048541009278847	-0.00044714705186636	0.0013748245556312	-0.000712135168113252	0.0016477226747629	0	0
elbow22	PSII	-0.00052005012531324	-0.00068056501808971	-0.0241004618168165	0.0240359823905704	-0.0311660547254018	0.0326140042048083	0	0
elbow15	PSII	-0.00085668717247663	-0.00076680800103428	-0.0110940669193567	0.00917861826597592	-0.014281100689971	0.0121335324869471	0	0
elbow05	PSII	0.00090909090909083	0.00092409376524840	-0.00090476880207508	0.00258900264894773	-0.00141364336287523	0.0031459777388260	0	0
elbow30	PSIa	-0.00104249284645657	-0.0012778953184661	-0.0659383353503506	0.0656609612462084	-0.0855536624066189	0.0885150601827155	0	0
elbow10	PSII	0.00110951687569244	0.0011871449268079	-0.0978012740250651	0.101531340852114	-0.128677069884458	0.131719610642969	0	0
elbow18	PSII	0.00113458097409205	0.00042960344821551	-0.07812309233905	0.0830111531312705	-0.101965575159898	0.106685172430412	0	0
elbow07	PSIa	0.00126262626262622	0.00125447999277994	-0.00115595771128142	0.00368641200139634	-0.00189145098812117	0.0044634856100236	0	0
elbow03	PSV	0.00154401154401175	0.0015964784304912	-0.0230261447620116	0.0252524353547185	-0.030111609579553	0.0328407172140487	0	0
elbow06	PSIa	0.00184275184275202	0.00184070311152699	-0.00168249573099555	0.00538649396411933	-0.00264907931558245	0.0064196073084816	0	0
elbow30	PSII	-0.0018553828665002	-0.00185043208295685	-0.0488014166649993	0.0448501024610332	-0.0621865661815629	0.0585805237596373	0	0
elbow07	PSV	0.00192722681359046	0.00198820533949921	-0.0239868482271311	0.0278874352556704	-0.0321503777791115	0.0363675108838208	0	0
elbow15	PSIb	0.00220959595959597	0.00222336804002774	-0.0101978238106322	0.0149557898081323	-0.0143337479123241	0.0186850980529172	0	0
elbow29	PSII	-0.00221104324511935	-0.0023453768767322	-0.0623444828076246	0.0589054594023438	-0.0803848486320499	0.0775399244787909	0	0
elbow11	PSIII	0.00267379679144385	0.00267374062219731	-0.00236695793423428	0.00788816200348681	-0.00389574857612204	0.0093958125728088	0	0
elbow14	PSV	0.00267379679144386	0.00267706789357653	-0.00244734860451049	0.00773878486007679	-0.00383318614274838	0.0092124509003560	0	0
elbow31	PSII	0.00269981075390453	0.00236408935424468	-0.101302299478061	0.105782497642674	-0.132265709404823	0.142099665410648	0	0
elbow14	PSIa	0.00284090909090907	0.00282773945858111	-0.00256894876971969	0.00840654004993065	-0.00419129550625583	0.0099725606558906	0	0
elbow22	PSIII	0.00287548903859058	0.00264433164638064	-0.0402765768224811	0.0470387887386465	-0.0545135544310753	0.0606215738828322	0	0
elbow09	PSII	0.00297619047619034	0.00298412123538537	-0.0134845480028968	0.0197025616997223	-0.0184149737563093	0.0247748710084571	0	0
elbow30	PSIII	0.00299586776859505	0.00303748366785688	-0.0457418766935958	0.0513857395076625	-0.0616753938877363	0.0659534516752862	0	0
elbow33	PSIII	0.00303030303030307	0.00305561151553908	-0.00275880723366494	0.00890176055702721	-0.00455606778983024	0.0106419960368761	0	0
elbow33	PSV	0.00331611570247927	0.0033298658817477	-0.0260497926599029	0.033141352252703	-0.0364450234644205	0.0429321567424798	0	0
elbow05	PSIb	0.00349650349650347	0.00353255278918301	-0.00328600022965457	0.010035063548881	-0.00529008907365536	0.0123001863767965	0	0
elbow11	PSII	-0.00353372434017594	-0.0035795028619778	-0.0130348414204964	0.00621985493383202	-0.0156895148979071	0.0090417485616536	0	0
elbow25	PSII	0.0038277511961723	0.00379890470792276	-0.00350279029476001	0.0110788188421384	-0.00559045113282623	0.0132077484254322	0	0
elbow23	PSIa	0.00397515206887845	0.00393662143659898	0.000626920095460139	0.00741711245318 4	-0.000458401528310861	0.0086130317221374	1	0
elbow03	PSIII	0.00413223140495864	0.00410309650839074	-0.00375477237741979	0.0121494890228649	-0.00624765777674245	0.014589541058429	0	0
elbow28	PSII	0.0044111378152125	0.00451022926722728	-0.0134915009768299	0.0220528093375507	-0.0189982377323745	0.0273561510404996	0	0
elbow19	PSIa	0.00452280228674301	0.00457668583412046	-0.00153188171128287	0.0105342889673481	-0.00336522832997142	0.0126062700556459	0	0
elbow19	PSV	-0.005	-0.00500912728711257	-0.0143076974084012	0.00406847627438736	-0.0176612003846256	0.0067371002651398	0	0
elbow32	PSIa	0.00501067341517255	0.00500963964123243	-0.0020272102781133	0.0119844785927089	-0.00410860735714855	0.0143952696765176	0	0
elbow16	PSIb	0.00505050505050505	0.00509418304554701	-0.0045793057259238	0.0147070477324004	-0.00777098838928628	0.0178154629152215	0	0
elbow23	PSIb	0.00505050505050505	0.00502224010273269	-0.00443732443843088	0.0146172176167119	-0.00748407170775788	0.0176962632109214	0	0
elbow18	PSIb	0.00505050505050506	0.00509001321846675	-0.00469020905574599	0.0147099020576005	-0.00775966423869812	0.0177401275708017	0	0
elbow17	PSII	0.00542699724517908	0.0054843514631355	-0.00114258676448865	0.0118388205605084	-0.0033430131066615	0.0140366863934024	0	0
elbow03	PSIb	0.00561868686868676	0.00572922945750138	-0.0130158522555559	0.0237187051781912	-0.0191694053583803	0.0296381096555069	0	0
elbow09	PSIb	0.00568181818181808	0.00570156977700069	-0.00515103294613404	0.0166886256597083	-0.00836744709994093	0.020204810794621	0	0
elbow08	PSV	0.00568181818181819	0.00570870689656877	-0.00515138402408402	0.0162666347480293	-0.00845147990948512	0.0198416139626872	0	0
elbow25	PSIa	0.00575232164799906	0.00574159167700049	-0.00166168728935898	0.0131793758746072	-0.00417025686009526	0.0155416427649207	0	0
elbow20	PSIII	0.0058618075329307	0.00534787110490819	-0.157295394618755	0.170189447940996	-0.20413806044395	0.22790092050609	0	0
elbow15	PSV	0.00603109012199924	0.00598260110243875	-0.00077800686267282	0.0129096913012711	-0.00284042842754717	0.0152210801066907	0	0
elbow19	PSII	0.00623179850452577	0.00619747092939639	-0.0108302659194331	0.0232678046971214	-0.0157037256159318	0.0289141146719326	0	0
elbow12	PSIII	0.00649350649350648	0.00653826204011345	-0.0059472048997847	0.0188494787389582	-0.00937589910724547	0.0227957897551866	0	0
elbow07	PSIII	0.00649350649350649	0.00659296975788493	-0.00601898186718304	0.0188541360783645	-0.00980851550954934	0.0229132494090983	0	0
elbow11	PSIa	0.00660633873091212	0.00666023227836503	-0.00444256345377063	0.017341815146621	-0.00771815944207531	0.0210555308488527	0	0
elbow10	PSV	-0.00703041393083252	-0.00712834279471148	-0.0757013057788884	0.0610870118462435	-0.0979106210164925	0.0823668888540564	0	0
elbow23	PSII	0.00722222222222224	0.00717440581881117	0.000516130791366882	0.0140520193324578	-0.0016445406078245	0.0160575278593774	1	0
elbow06	PSV	0.00757575757575741	0.0076789154043771	-0.00700545242819516	0.0219287145132119	-0.0114309489163429	0.0265140463143367	0	0
elbow28	PSIII	0.00757575757575761	0.00759718469460701	-0.00725558864863004	0.0220357521799915	-0.0118112723085827	0.0266437006960717	0	0
elbow04	PSV	-0.00781250000000008	-0.00781228396854854	-0.0222575258392644	0.0064814808940771	-0.0262614486071374	0.0110870123728287	0	0
elbow27	PSII	-0.00814241571225679	-0.00832050275373305	-0.0480429402529088	0.0324410436971774	-0.059542073413319	0.0450933482887677	0	0
elbow29	PSIb	-0.00818278943278941	-0.00809318043639618	-0.0422904354333266	0.0265947676858421	-0.0530402118191731	0.0376806575123965	0	0
elbow18	PSIa	0.00884905184338435	0.00903134523854146	-0.031600363376878	0.0483439144187124	-0.045005050674939	0.0608900027626782	0	0
elbow16	PSV	-0.0089503910068426	-0.00898964789981955	-0.0279747631237508	0.010391195150418	-0.0345107952126809	0.0164256902279455	0	0
elbow32	PSIII	0.00909090909090909	0.00920162333558235	-0.00819971557748205	0.0263120711634111	-0.0140730374614541	0.0322334452967591	0	0
elbow27	PSV	0.00926332382424696	0.00959820199502028	-0.0397719226201089	0.0577952222319576	-0.0568832813207396	0.0727676045442617	0	0
elbow31	PSIII	-0.00926540037502602	-0.00913935962521129	-0.0564298249821211	0.0375617441816082	-0.0713654951526476	0.0531457268098275	0	0
elbow28	PSV	0.00940031530439004	0.00956964673582372	-0.0249812856121887	0.0429617335325242	-0.0345401726016496	0.0543595866305961	0	0
elbow02	PSIII	-0.00984848484848486	-0.0102079691977347	-0.0637029535990015	0.0460681108756409	-0.0806668772346348	0.0615139412881279	0	0
elbow20	PSV	-0.0099730399714594	-0.00900700389906101	-0.159303969404843	0.134954908984162	-0.206853502073285	0.18142783924479	0	0

cluster	type	obs_estimate	unbiased_estimate	ci_limit_0.025	ci_limit_0.975	ci.adj_limit_0.005	ci.adj_limit_0.995	signif_ci	signif_ci.adj
elbow15	PSIa	0.0100509340483743	0.0101020859302986	0.00217573646574286	0.0180325952735333	-0.000325854210878307	0.0204782127319348	1	0
elbow16	PSIa	0.0113207607942007	0.0114959014827861	-0.00362505517379573	0.025927695407413	-0.00812235149611184	0.030243507797159	0	0
elbow06	PSIII	0.0113636363636364	0.0114429980088675	-0.0108320484703715	0.0331426153084382	-0.0189315999138023	0.0399211324997518	0	0
elbow14	PSIb	0.0116792929292929	0.0114916244905993	-0.0184407497510255	0.0413060230918711	-0.0270967777985296	0.0496319077429995	0	0
elbow08	PSIII	0.0117079889807163	0.0117773928125343	-0.00439574582975589	0.0277774993261194	-0.00922522462209594	0.0328616750649626	0	0
elbow16	PSII	-0.0120059446136001	-0.0120088459508138	-0.0331449004112008	0.0093912351170274	-0.0397354868216515	0.0162496604414524	0	0
elbow04	PSIb	0.012310606060606	0.0119820737882075	-0.0352826346127417	0.0607465167787233	-0.0474840770541834	0.0758194306034998	0	0
elbow08	PSII	0.0123295821825234	0.012253257364117	-0.00968110852843805	0.034995043442348	-0.0161165523166152	0.0424505201137123	0	0
elbow16	PSIII	-0.0125095492742552	-0.0127678864366297	-0.0468928912265474	0.021981495087175	-0.0583887088824994	0.0315585321589158	0	0
elbow08	PSIa	0.013035763035763	0.0130589134869979	-0.00450689190283913	0.0306444065745184	-0.00997954164359583	0.0359675569717274	0	0
elbow12	PSIb	0.0132575757575757	0.0133247709220772	-0.00437941138382035	0.0310479533587282	-0.0102687293578308	0.0369266112382182	0	0
elbow29	PSIa	0.0132656778524677	0.0135741898428905	-0.0312927975336803	0.0569633618078306	-0.043583922352273	0.0704045283048104	0	0
elbow32	PSIb	-0.0138888888888889	-0.0137148796229482	-0.033022658450835	0.00551974792617395	-0.0388878597058413	0.0118074265691454	0	0
elbow07	PSII	0.0141754915775961	0.014063885632385	0.000538846321955205	0.0280206962404282	-0.0034967068504453	0.0324314539589351	1	0
elbow32	PSII	-0.0150282173966384	-0.0149431816022926	-0.0524512662179059	0.0217931035955608	-0.0621048336402045	0.0330661336095266	0	0
elbow04	PSII	-0.0150421508316246	-0.0151360546996404	-0.0510642721504587	0.0219385618921792	-0.0624061886199936	0.0323607911539556	0	0
elbow19	PSIII	0.0151515151515152	0.0151634200252882	-0.0139619323514568	0.0437097238917713	-0.0235349311809865	0.0532671226776654	0	0
elbow25	PSIII	0.0151515151515152	0.015127637055988	-0.0139446430937931	0.0439083716820574	-0.0221382374054423	0.0534018823488622	0	0
elbow26	PSII	0.0152325027444642	0.0151187294060425	0.00304147805059231	0.0275439109875327	-0.000759946634369676	0.031297689693736	1	0
elbow31	PSIb	0.0161227661227662	0.0159363578802243	-0.0348504371857421	0.0686469139845903	-0.0515569695666345	0.0858327600173688	0	0
elbow05	PSV	-0.0162878787878788	-0.0161642698255927	-0.0559077010673077	0.0232562511056772	-0.0676334871476231	0.0347908677460851	0	0
elbow25	PSIb	-0.0183080808080809	-0.0176863656866609	-0.0651838035943434	0.0267539330979035	-0.0781842406074495	0.0399173536452364	0	0
elbow22	PSV	-0.0183841888194894	-0.0178706983602927	-0.0785945588818943	0.0419007264514471	-0.0967075109533518	0.0596293218177502	0	0
elbow01	PSV	-0.018767217630854	-0.018785790323049	-0.0567974405461299	0.019886886611676	-0.0680191097818442	0.0326258477912702	0	0
elbow15	PSIII	0.018904958677686	0.0188167340076127	-0.0023512093166005	0.0401895003178492	-0.00787550827247413	0.0463398215560841	0	0
elbow14	PSIII	0.0191558441558442	0.0192646071527299	-0.00386562080093405	0.0421403722696757	-0.0119614218454937	0.0496237310925823	0	0
elbow12	PSII	0.0194434039958337	0.019457364905652	0.00296285831285668	0.0359013836159515	-0.00212227750135941	0.0411419331497941	1	0
elbow29	PSIII	-0.0197314049586776	-0.0198418508242925	-0.10876002309480 4	0.0695912803165046	-0.135177556469368	0.0991499228936913	0	0
elbow02	PSIb	-0.0198378010878011	-0.0199239295815698	-0.0636510364996723	0.0243190858237554	-0.0774231778552472	0.0383788917559244	0	0
elbow17	PSIa	-0.0202274212512638	-0.0202903992273477	-0.081375124624074	0.0419693757251012	-0.101062734068124	0.0630050361725613	0	0
elbow13	PSV	0.0203389891645388	0.0200673178330646	-0.0308866840708712	0.0728813111264476	-0.0459191344509512	0.0874230496358414	0	0
elbow24	PSIb	0.0205419580419582	0.0205523994322303	-0.0485861046112491	0.0902344926827892	-0.0690114638630405	0.113520090402148	0	0
elbow01	PSIII	-0.0208333333333333	-0.0207501080275584	-0.0594724009894258	0.0178001489848489	-0.0712180753530306	0.0302647580928263	0	0
elbow23	PSV	-0.0212609970674487	-0.0215747026707289	-0.0625786883752324	0.0205093699130561	-0.0745641628463315	0.0323111214867129	0	0
elbow13	PSIII	-0.0226948630691946	-0.022517622536326	-0.0866030488365028	0.0407030484353799	-0.105065299450094	0.0605168032755831	0	0
elbow23	PSIII	-0.0227272727272727	-0.022113631303798	-0.104283745365882	0.0567871946658009	-0.130533538091497	0.0841123700897731	0	0
elbow28	PSIa	-0.0227652915174628	-0.0225533407608992	-0.119732958403972	0.0735970314359325	-0.153071566558143	0.101450968368973	0	0
elbow26	PSIa	0.0233584429562743	0.0233645619142245	0.00321262668516409	0.0430822419396865	-0.00212197839025583	0.0492643177274281	1	0
elbow30	PSV	-0.023615225441414	-0.0236850437457532	-0.0621556993615516	0.0160413966206164	-0.074827109267484	0.0287062074040567	0	0
elbow22	PSIb	-0.0243055555555556	-0.0247575896082529	-0.0855775859770814	0.0365003149433325	-0.103327400861344	0.0539526434515306	0	0
elbow04	PSIII	-0.025	-0.0253020678358841	-0.0710250738153131	0.0207631064281267	-0.0861416727163214	0.0347999905116192	0	0
elbow26	PSIII	0.0259740259740257	0.0259616564684058	-0.0035789351410119	0.0565670744708047	-0.0131219741717421	0.0651314989703958	0	0
elbow30	PSIb	0.0261606449106449	0.0255412398203124	-0.0456608257213078	0.0975436006323735	-0.0677385293494299	0.119599004104006	0	0
elbow03	PSII	-0.0272760346614337	-0.0269971817943186	-0.0946412610742562	0.0389318134875973	-0.117225258051896	0.0582634121900053	0	0
elbow12	PSIa	0.0275887362631695	0.0275793492567401	-0.00259031784936769	0.0583493300297583	-0.0118788892980751	0.0668653671295504	0	0
elbow27	PSIb	0.0277777777777777	0.0279544913231194	-0.0165389531059476	0.0708304723439572	-0.0304138309644926	0.0851557712899998	0	0
elbow24	PSII	0.0282668089235424	0.0282734524872374	0.00458122212805128	0.0521352809852738	-0.00398846469964789	0.0601108059430522	1	0
elbow27	PSIa	-0.0307803232431645	-0.0308637423604223	-0.128486660889513	0.0676261422928788	-0.159811888998312	0.0975962349242502	0	0
elbow19	PSIb	0.0315656565656566	0.031886736586328	-0.0572452236198576	0.118849631639955	-0.0877729429708554	0.144284833702755	0	0
elbow13	PSIa	0.0322744440300675	0.0323182453099227	-0.0236832477858196	0.0882502270660854	-0.040894362166181	0.10496561361099	0	0
elbow02	PSII	-0.0341864305949235	-0.0341710257297277	-0.0786662538436544	0.0101291627402343	-0.0930936727131151	0.0235775730662045	0	0
elbow18	PSV	0.0352462488003754	0.0351738681343908	0.000181267821958148	0.0701845511442362	-0.01022791598117	0.0816217680730943	1	0
elbow33	PSIa	0.0359986615663316	0.0358696793711584	0.00482731314708232	0.0671763399440162	-0.00577713585967439	0.0768163112976923	1	0
elbow32	PSV	-0.0391277673350042	-0.040142788659445	-0.107096703583476	0.0311622244212954	-0.129862607495888	0.0511491629693295	0	0
elbow33	PSII	0.0396445659603555	0.0398522048977806	-0.0332662982781187	0.112082062398873	-0.0575504939529457	0.13417394677016	0	0
elbow09	PSIII	-0.0398268398268398	-0.0397674070663457	-0.0932709955061425	0.0144530925346463	-0.109408113123053	0.0310825630934072	0	0
elbow20	PSII	0.0438799597652699	0.0429996791389032	-0.0384685367756634	0.128494098633033	-0.0635422198404513	0.155420849077802	0	0
elbow24	PSV	0.044290474992778	0.0439282693105675	-0.0279672782636496	0.115487367120846	-0.0490631599447994	0.140730294161557	0	0
elbow13	PSII	0.0449175927649822	0.0450361991168396	0.00219346230204555	0.0855437324448897	-0.0100114770758752	0.0999774422699198	1	0
elbow10	PSIb	0.0482566045066045	0.047753871976538	-0.0300010503507835	0.126200996880541	-0.0544698466990553	0.150135727859157	0	0
elbow08	PSIb	0.048951048951049	0.0479640393106262	-0.0336861052903406	0.135523406883805	-0.0630203799710996	0.163164212796809	0	0
elbow02	PSV	-0.0504545454545455	-0.0503689363649102	-0.133923703171053	0.0324799521677665	-0.161533003444324	0.0589849567499375	0	0
elbow33	PSIb	0.0505050505050505	0.0499305652003921	-0.0348666450954271	0.138079474703876	-0.0656403702065573	0.164606877024563	0	0
elbow13	PSIb	0.0505293317793318	0.0505090958224124	-0.00900449582542516	0.110183666629398	-0.0253899902452369	0.128174002798385	0	0
elbow21	PSIb	0.0514374514374515	0.0507227197551403	-0.0859270979687797	0.194433235520456	-0.134681338706186	0.238197275418701	0	0
elbow25	PSV	-0.0523735733963007	-0.0520805162556627	-0.112766502344584	0.00918977667694684	-0.133543905928864	0.0260883906480636	0	0
elbow21	PSIII	0.0544103178461468	0.0543980936953206	-0.000198112581770682	0.110336409082272	-0.018285351049264	0.128419840102827	0	0
elbow26	PSIb	-0.0565656565656565	-0.0566467258436847	-0.233356978155262	0.115227502625693	-0.284095661291464	0.171313025856074	0	0
elbow05	PSIa	-0.0591040716040717	-0.0595623071551925	-0.174016027344068	0.0551920696020131	-0.212795550874357	0.0921487708545622	0	0
elbow27	PSIII	-0.0600229761326019	-0.059786943739875	-0.16398124450224	0.0416143453925644	-0.195048243324607	0.0737215001908556	0	0
elbow07	PSIb	-0.0624999999999999	-0.0619475341587087	-0.177766354992006	0.0499006060633896	-0.218341710888638	0.0896303980231575	0	0
elbow21	PSIa	0.0666156498575894	0.0660729959633815	-0.00798397017027392	0.143641400323724	-0.0347378385583422	0.166164609326389	0	0
elbow10	PSIII	0.0671733569460844	0.0668270668874155	-0.0357125137615257	0.172389418519694	-0.0758725398512603	0.202674489820909	0	0
elbow04	PSIa	-0.0681786735541002	-0.0679898230317469	-0.187681793584891	0.0503988286080355	-0.221472161909834	0.0834354418198451	0	0
elbow03	PSIa	-0.0693575595452732	-0.0693925230563371	-0.223357179743501	0.0830425238172113	-0.270753944346763	0.130901820418748	0	0
elbow21	PSV	0.0697862039753321	0.0697692200519932	0.0145767218494161	0.124956719724361	-0.00103836205180636	0.141846328983436	1	0
elbow18	PSIII	0.081147128272797	0.0806424493357246	-0.00982133801625554	0.175464276324435	-0.0402745971506599	0.203996533299361	0	0
elbow29	PSV	-0.0820922478562204	-0.0823268242557697	-0.1731318167531	0.00954947749568932	-0.202781919562713	0.0380569631408318	0	0
elbow10	PSIa	-0.0901265118914986	-0.0907986830296714	-0.200277357065606	0.0193614064810138	-0.23400762592694	0.0556247160396554	0	0
elbow20	PSIb	0.0918803418803421	0.0915975360296477	-0.0321694807266479	0.215824316618756	-0.0671184013607661	0.252804874960677	0	0
elbow02	PSIa	-0.0981180466589231	-0.0982931046889943	-0.27073150032894	0.0723806980737637	-0.325868399231424	0.124247682125091	0	0
elbow09	PSIa	-0.107702285389999	-0.107131436862043	-0.260491647639119	0.0414555935943426	-0.306882841202082	0.0856793959346072	0	0

cluster	type	obs_estimate	unbiased_estimate	ci_limit_0.025	ci_limit_0.975	ci.adj_limit_0.005	ci.adj_limit_0.995	signif_ci	signif_ci.adj
elbow24	PSIII	0.121096661805218	0.121062630324984	0.00483679976342845	0.240106751098699	-0.0334436520764023	0.277252922575671	1	0
elbow21	PSII	-0.146346963122436	-0.147442088880481	-0.308710021635754	0.0170957211671334	-0.365148267857943	0.0679277401279606	0	0

Supplementary table 5. Statistical results from linear regression model bootstrapping for GBS phenotype and population analysis. The observed (obs_estimate) and estimated (unbiased_estimate) difference in proportions of clusters among total B cells for a given serotype-specificity and donor population (South African- over Dutch donors) were generated with a two-sided linear regression model with wild bootstrap simulation (9999 resamples). Confidence intervals (ci) and Bonferroni’s method adjusted ci (ci.adj) are shown, as well as statistical significance (signif_ci/ signif_ci.adj = 1).
