# Supplementary material for: Combinatorial multimer staining and spectral flow cytometry facilitate quantification and characterization of polysaccharide-specific B cell immunity
Source: Commun Biol. 2023 Oct 28;6:1095. doi: 10.1038/s42003-023-05444-3 (PMC10613281; doi:10.1038/s42003-023-05444-3)
Supplement: Supplementary file 3 — Description of Additional Supplementary Files [file 42003_2023_5444_MOESM3_ESM.pdf]

Description of Additional Supplementary Files

File name: Supplementary Data 1
Description: Source data behind the graphs in the figures.

File name: Supplementary Data 2
Description: Statistical results from linear regression model bootstrapping for Spn cross-sectional phenotype and serotype analysis. The observed (obs_estimate) and estimated (unbiased_estimate) proportions of clusters among total B cells for a given serotype-specificity and sample, were generated with an upper-sided linear regression model with wild bootstrap simulation (9999 resamples). Confidence intervals (ci) and Bonferroni's method adjusted ci (ci.adj) are shown, as well as statistical significance (signif_ci/ signif_ci.adj = 1).
