# Supplementary material for: Combinatorial multimer staining and spectral flow cytometry facilitate quantification and characterization of polysaccharide-specific B cell immunity
Source: Commun Biol. 2023 Oct 28;6:1095. doi: 10.1038/s42003-023-05444-3 (PMC10613281; doi:10.1038/s42003-023-05444-3)
Supplement: Supplementary file 5 — Supplementary Data 2 [file 42003_2023_5444_MOESM5_ESM.pdf]

| Supplementary data 2 | | | | | | | |
|---|---|---|---|---|---|---|---|
| cluster | type | obs_estimate | unbiased_estimate | ci_limit_0.05 | ci.adj_limit_0.00357142857142857 | signif_ci | signif_ci.adj |
| elbow02 | PS3 | 0,049868808 | 0,050062022 | 0,023916796 | 0,007442109 | 1 | 1 |
| elbow03 | PS19F | 0,108454984 | 0,10828408 | 0,049139345 | 0,012581391 | 1 | 1 |
| elbow12 | PS19F | 0,028202472 | 0,028180124 | 0,015238202 | 0,006695311 | 1 | 1 |
| elbow22 | PS3 | 0,072066764 | 0,072143482 | 0,048592785 | 0,033406243 | 1 | 1 |
| elbow24 | PS3 | 0,023295111 | 0,023248339 | 0,012181427 | 0,005125825 | 1 | 1 |
| elbow24 | PS1 | 0,035488191 | 0,035559035 | 0,015258346 | 0,00296391 | 1 | 1 |
| elbow02 | PS14 | 0,063593536 | 0,063777471 | 0,008077162 | -0,027667168 | 1 | 0 |
| elbow03 | PS15B | 0,125187621 | 0,125986717 | 0,02723063 | -0,034114835 | 1 | 0 |
| elbow03 | PS7F | 0,080551301 | 0,080795597 | 0,018086978 | -0,022874351 | 1 | 0 |
| elbow03 | PS9V | 0,113897479 | 0,113303016 | 0,032383169 | -0,018956291 | 1 | 0 |
| elbow04 | PS14 | 0,116961197 | 0,116411835 | 0,012978791 | -0,05535761 | 1 | 0 |
| elbow04 | PS23F | 0,151695407 | 0,150736944 | 0,004693672 | -0,08341464 | 1 | 0 |
| elbow06 | PS6B | 0,05642263 | 0,05617144 | 0,001629459 | -0,031792753 | 1 | 0 |
| elbow07 | PS3 | 0,020844488 | 0,020567777 | 0,000304645 | -0,012431635 | 1 | 0 |
| elbow08 | PS3 | 0,027177645 | 0,02716793 | 0,00358492 | -0,010910642 | 1 | 0 |
| elbow08 | PS1 | 0,055944928 | 0,056253144 | 0,012200062 | -0,013896332 | 1 | 0 |
| elbow09 | PS3 | 0,011284919 | 0,011325884 | 0,003401641 | -0,001023324 | 1 | 0 |
| elbow11 | PS15B | 0,04261636 | 0,04277258 | 0,00278086 | -0,022983941 | 1 | 0 |
| elbow11 | PS1 | 0,041879075 | 0,042085738 | 0,015308652 | -0,001136055 | 1 | 0 |
| elbow12 | PS9V | 0,026058373 | 0,025934497 | 0,002873525 | -0,013405547 | 1 | 0 |
| elbow15 | PS6B | 0,025894977 | 0,025879619 | 0,003921525 | -0,010208267 | 1 | 0 |
| elbow16 | PS1 | 0,04441956 | 0,044269971 | 0,01507113 | -0,00394438 | 1 | 0 |
| elbow21 | PS4 | 0,16336651 | 0,163612242 | 0,055853611 | -0,012689965 | 1 | 0 |
| elbow22 | PS1 | 0,035493897 | 0,035464321 | 0,010831155 | -0,006370062 | 1 | 0 |
| elbow25 | PS6B | 0,029481139 | 0,029434947 | 0,001684514 | -0,015323105 | 1 | 0 |
| elbow27 | PS3 | 0,008515867 | 0,008528769 | 0,000366625 | -0,004924165 | 1 | 0 |
| elbow28 | PS6B | 0,027595813 | 0,027443715 | 0,001744985 | -0,014050952 | 1 | 0 |
| elbow31 | PS3 | 0,018223097 | 0,018287247 | 7,79877E-06 | -0,011587024 | 1 | 0 |
| elbow33 | PS6B | 0,025390256 | 0,025463697 | 0,000736891 | -0,016333152 | 1 | 0 |
| elbow35 | PS18C | 0,018226209 | 0,018408384 | 0,000652471 | -0,010474755 | 1 | 0 |
| elbow35 | PS6B | 0,044950402 | 0,045013448 | 0,002080034 | -0,025259934 | 1 | 0 |
| elbow01 | PS14 | 0,000901005 | 0,001147537 | -0,020697606 | -0,033079483 | 0 | 0 |
| elbow01 | PS15B | -0,007974404 | -0,008043955 | -0,019457233 | -0,027425566 | 0 | 0 |
| elbow01 | PS18C | -0,009044279 | -0,009135255 | -0,020767817 | -0,028431849 | 0 | 0 |
| elbow01 | PS19A | -0,013017286 | -0,01295709 | -0,021408072 | -0,026329184 | 0 | 0 |
| elbow01 | PS19F | 0,002932769 | 0,003084169 | -0,010630012 | -0,018191137 | 0 | 0 |
| elbow01 | PS23F | -0,020873959 | -0,020873388 | -0,028461732 | -0,033605191 | 0 | 0 |
| elbow01 | PS3 | 0,010438207 | 0,010508117 | -0,002686698 | -0,010389943 | 0 | 0 |
| elbow01 | PS4 | -0,017158296 | -0,017237891 | -0,023531908 | -0,027702311 | 0 | 0 |
| elbow01 | PS5 | 0,020334832 | 0,020306564 | -0,01645003 | -0,039669025 | 0 | 0 |
| elbow01 | PS6A | 0,028807395 | 0,02873598 | -0,00048294 | -0,018194832 | 0 | 0 |
| elbow01 | PS6B | 0,01789283 | 0,017936733 | -0,009840044 | -0,028777601 | 0 | 0 |
| elbow01 | PS7F | 0,000362533 | 0,000592495 | -0,017356755 | -0,028862615 | 0 | 0 |
| elbow01 | PS9V | -0,006490084 | -0,006521141 | -0,019913019 | -0,028332241 | 0 | 0 |
| elbow01 | PS1 | -0,007111262 | -0,007083378 | -0,016040496 | -0,022196141 | 0 | 0 |
| elbow02 | PS15B | -0,025911244 | -0,025796493 | -0,044182458 | -0,056899456 | 0 | 0 |
| elbow02 | PS18C | -0,023961212 | -0,024030492 | -0,044694655 | -0,058212191 | 0 | 0 |
| elbow02 | PS19A | 0,011036481 | 0,011207577 | -0,020718353 | -0,038857407 | 0 | 0 |
| elbow02 | PS19F | -0,036804072 | -0,036966565 | -0,054739475 | -0,067364078 | 0 | 0 |
| elbow02 | PS23F | -0,063339966 | -0,063376072 | -0,074967205 | -0,081599438 | 0 | 0 |
| elbow02 | PS4 | -0,013101118 | -0,012966148 | -0,034821354 | -0,047262649 | 0 | 0 |
| elbow02 | PS5 | -0,013989316 | -0,014100181 | -0,04468782 | -0,063453695 | 0 | 0 |
| elbow02 | PS6A | 0,063600679 | 0,063740045 | -0,005786381 | -0,049746093 | 0 | 0 |
| elbow02 | PS6B | -0,004792957 | -0,004849483 | -0,040827991 | -0,064708661 | 0 | 0 |
| elbow02 | PS7F | 0,017070649 | 0,017032449 | -0,016924516 | -0,036880843 | 0 | 0 |
| elbow02 | PS9V | -0,027182172 | -0,027131064 | -0,054073359 | -0,071568267 | 0 | 0 |
| elbow02 | PS1 | 0,003911903 | 0,003976638 | -0,021976161 | -0,03735796 | 0 | 0 |
| elbow03 | PS14 | -0,037346394 | -0,036636439 | -0,087502264 | -0,119398686 | 0 | 0 |
| elbow03 | PS18C | 0,068065697 | 0,068589569 | -0,002420526 | -0,048884909 | 0 | 0 |
| elbow03 | PS19A | 0,087481012 | 0,086767321 | -0,008344757 | -0,068786831 | 0 | 0 |
| elbow03 | PS23F | -0,206431621 | -0,206562816 | -0,234966237 | -0,2523294 | 0 | 0 |
| elbow03 | PS3 | -0,071480667 | -0,071761235 | -0,116168307 | -0,142878119 | 0 | 0 |
| elbow03 | PS4 | 0,016992575 | 0,016202454 | -0,093288933 | -0,164037279 | 0 | 0 |
| elbow03 | PS5 | -0,02505189 | -0,024883156 | -0,091102434 | -0,1379007 | 0 | 0 |
| elbow03 | PS6A | -0,026618809 | -0,026650507 | -0,081939344 | -0,116005056 | 0 | 0 |
| elbow03 | PS6B | -0,15963675 | -0,159828785 | -0,200807383 | -0,228436331 | 0 | 0 |
| elbow03 | PS1 | -0,074064538 | -0,074264844 | -0,15311025 | -0,200691639 | 0 | 0 |
| elbow04 | PS15B | 0,00048713 | 0,000488477 | -0,053122303 | -0,083367887 | 0 | 0 |
| elbow04 | PS18C | 0,038006652 | 0,038346971 | -0,025072073 | -0,059826688 | 0 | 0 |
| elbow04 | PS19A | -0,002010938 | -0,001894002 | -0,06821266 | -0,113852939 | 0 | 0 |

| cluster | type | obs_estimate | unbiased_estimate | ci_limit_0.05 | ci.adj_limit_0.00357142857142857 | signif_ci | signif_ci.adj |
|---|---|---|---|---|---|---|---|
| elbow04 | PS19F | -0,029048317 | -0,028861634 | -0,076827498 | -0,106794692 | 0 | 0 |
| elbow04 | PS3 | -0,126517641 | -0,126401009 | -0,152805962 | -0,169394463 | 0 | 0 |
| elbow04 | PS4 | -0,04458752 | -0,045099033 | -0,13778095 | -0,193797105 | 0 | 0 |
| elbow04 | PS5 | 0,050357855 | 0,050194771 | -0,020845309 | -0,071961253 | 0 | 0 |
| elbow04 | PS6A | -0,006392878 | -0,006204533 | -0,080935996 | -0,124493725 | 0 | 0 |
| elbow04 | PS6B | -0,136216681 | -0,136385617 | -0,191760845 | -0,230345135 | 0 | 0 |
| elbow04 | PS7F | 0,082994863 | 0,083506712 | -0,029042634 | -0,101896273 | 0 | 0 |
| elbow04 | PS9V | 0,015536263 | 0,016031396 | -0,039673278 | -0,073288816 | 0 | 0 |
| elbow04 | PS1 | -0,111265392 | -0,111556424 | -0,165593866 | -0,203649374 | 0 | 0 |
| elbow05 | PS14 | -0,001477383 | -0,001440723 | -0,005913477 | -0,008480889 | 0 | 0 |
| elbow05 | PS15B | 0,005521711 | 0,005501013 | -0,001986589 | -0,006363639 | 0 | 0 |
| elbow05 | PS18C | 0,022279066 | 0,0222205 | -0,012994016 | -0,033552769 | 0 | 0 |
| elbow05 | PS19A | -0,001710483 | -0,001726122 | -0,005862331 | -0,008539453 | 0 | 0 |
| elbow05 | PS19F | 0,002514459 | 0,002553835 | -0,004326887 | -0,008973913 | 0 | 0 |
| elbow05 | PS23F | -0,004041485 | -0,00403857 | -0,00746755 | -0,009601222 | 0 | 0 |
| elbow05 | PS3 | -0,002225246 | -0,002209179 | -0,006307889 | -0,008854296 | 0 | 0 |
| elbow05 | PS4 | -0,004041485 | -0,004059163 | -0,007419129 | -0,009501869 | 0 | 0 |
| elbow05 | PS5 | -0,004041485 | -0,004023638 | -0,00749648 | -0,009747717 | 0 | 0 |
| elbow05 | PS6A | -0,004041485 | -0,004024713 | -0,007472986 | -0,009449171 | 0 | 0 |
| elbow05 | PS6B | -0,004041485 | -0,004028936 | -0,007460708 | -0,009488397 | 0 | 0 |
| elbow05 | PS7F | -0,003480003 | -0,003521749 | -0,006747566 | -0,008792659 | 0 | 0 |
| elbow05 | PS9V | -0,001388965 | -0,001407292 | -0,006822191 | -0,01042774 | 0 | 0 |
| elbow05 | PS1 | 0,000174269 | 0,000212323 | -0,004927899 | -0,008037252 | 0 | 0 |
| elbow06 | PS14 | 0,005611089 | 0,005748865 | -0,018375215 | -0,033182685 | 0 | 0 |
| elbow06 | PS15B | 0,013983793 | 0,014018852 | -0,011278689 | -0,027584296 | 0 | 0 |
| elbow06 | PS18C | 0,002523045 | 0,002444873 | -0,017613374 | -0,031213071 | 0 | 0 |
| elbow06 | PS19A | -0,009331098 | -0,009400145 | -0,029648596 | -0,042491577 | 0 | 0 |
| elbow06 | PS19F | -0,004019559 | -0,004246017 | -0,020845567 | -0,032496299 | 0 | 0 |
| elbow06 | PS23F | -0,035056191 | -0,035091967 | -0,043313222 | -0,048900279 | 0 | 0 |
| elbow06 | PS3 | -0,012284875 | -0,012301109 | -0,025665342 | -0,034690275 | 0 | 0 |
| elbow06 | PS4 | 0,010395168 | 0,010361932 | -0,015371496 | -0,031263065 | 0 | 0 |
| elbow06 | PS5 | 0,041766986 | 0,041510363 | -0,018638287 | -0,05458044 | 0 | 0 |
| elbow06 | PS6A | -0,016581743 | -0,016527269 | -0,034662192 | -0,047731774 | 0 | 0 |
| elbow06 | PS7F | -0,023556288 | -0,023596789 | -0,033434701 | -0,03960302 | 0 | 0 |
| elbow06 | PS9V | -0,010164704 | -0,010156059 | -0,022951646 | -0,031196173 | 0 | 0 |
| elbow06 | PS1 | -0,019708255 | -0,019748093 | -0,033505203 | -0,042902762 | 0 | 0 |
| elbow07 | PS14 | 0,00256465 | 0,002336404 | -0,025794984 | -0,043169677 | 0 | 0 |
| elbow07 | PS15B | -0,008545024 | -0,008677797 | -0,026016297 | -0,037352804 | 0 | 0 |
| elbow07 | PS18C | -0,018138043 | -0,018244491 | -0,032821939 | -0,041349064 | 0 | 0 |
| elbow07 | PS19A | 0,007204499 | 0,007556201 | -0,018545119 | -0,034496209 | 0 | 0 |
| elbow07 | PS19F | 0,012762532 | 0,012715004 | -0,006325608 | -0,018000211 | 0 | 0 |
| elbow07 | PS23F | -0,018994496 | -0,018709491 | -0,050552217 | -0,070528604 | 0 | 0 |
| elbow07 | PS4 | 0,030982123 | 0,030890567 | -0,016698841 | -0,049382337 | 0 | 0 |
| elbow07 | PS5 | 0,002101075 | 0,00244554 | -0,035639975 | -0,058468552 | 0 | 0 |
| elbow07 | PS6A | -0,018445045 | -0,018366365 | -0,039863871 | -0,05334391 | 0 | 0 |
| elbow07 | PS6B | -0,032308105 | -0,032262012 | -0,046712747 | -0,056533857 | 0 | 0 |
| elbow07 | PS7F | 0,01245204 | 0,012474625 | -0,022414939 | -0,043940004 | 0 | 0 |
| elbow07 | PS9V | 0,007973694 | 0,008095349 | -0,007443563 | -0,017564322 | 0 | 0 |
| elbow07 | PS1 | -0,000454387 | -0,000520707 | -0,01885771 | -0,030496452 | 0 | 0 |
| elbow08 | PS14 | 0,018376802 | 0,018447093 | -0,006176979 | -0,021856423 | 0 | 0 |
| elbow08 | PS15B | -0,008298829 | -0,008293921 | -0,025386867 | -0,036778548 | 0 | 0 |
| elbow08 | PS18C | -0,002447435 | -0,002457699 | -0,019798989 | -0,031026903 | 0 | 0 |
| elbow08 | PS19A | 0,009140999 | 0,009200356 | -0,011250487 | -0,024074735 | 0 | 0 |
| elbow08 | PS19F | -0,029891108 | -0,0299162 | -0,038823994 | -0,043889401 | 0 | 0 |
| elbow08 | PS23F | -0,022961038 | -0,022881697 | -0,037602134 | -0,047252463 | 0 | 0 |
| elbow08 | PS4 | 0,006428624 | 0,0063339 | -0,027814294 | -0,047929876 | 0 | 0 |
| elbow08 | PS5 | -0,021462536 | -0,021449471 | -0,036660952 | -0,04679275 | 0 | 0 |
| elbow08 | PS6A | 0,001927655 | 0,001871657 | -0,015057971 | -0,02567728 | 0 | 0 |
| elbow08 | PS6B | 0,009517595 | 0,009678141 | -0,020617023 | -0,0402241 | 0 | 0 |
| elbow08 | PS7F | -0,024267933 | -0,024140826 | -0,032419047 | -0,037158473 | 0 | 0 |
| elbow08 | PS9V | -0,01918537 | -0,019154263 | -0,032857074 | -0,041496432 | 0 | 0 |
| elbow09 | PS14 | 0,000151337 | 0,000172221 | -0,00853391 | -0,013847202 | 0 | 0 |
| elbow09 | PS15B | -0,005309468 | -0,005324935 | -0,010927894 | -0,014299956 | 0 | 0 |
| elbow09 | PS18C | 0,00137068 | 0,001374834 | -0,007145024 | -0,012542002 | 0 | 0 |
| elbow09 | PS19A | -0,002708298 | -0,002758156 | -0,010089207 | -0,014420954 | 0 | 0 |
| elbow09 | PS19F | 0,003069679 | 0,003061351 | -0,007407522 | -0,013422628 | 0 | 0 |
| elbow09 | PS23F | -0,008805972 | -0,008840488 | -0,012416569 | -0,014890869 | 0 | 0 |
| elbow09 | PS4 | -0,004647967 | -0,004710162 | -0,011323807 | -0,015569353 | 0 | 0 |
| elbow09 | PS5 | -0,008805972 | -0,008796998 | -0,012556398 | -0,015029666 | 0 | 0 |
| elbow09 | PS6A | 0,010269824 | 0,010333657 | -0,007938609 | -0,019776513 | 0 | 0 |
| elbow09 | PS6B | 0,009356422 | 0,009272434 | -0,007607565 | -0,018008727 | 0 | 0 |

| cluster | type | obs_estimate | unbiased_estimate | ci_limit_0.05 | ci.adj_limit_0.00357142857142857 | signif_ci | signif_ci.adj |
|---|---|---|---|---|---|---|---|
| elbow09 | PS7F | -0,001098977 | -0,001144593 | -0,006966958 | -0,011163311 | 0 | 0 |
| elbow09 | PS9V | -0,006641925 | -0,006612058 | -0,010213339 | -0,012369475 | 0 | 0 |
| elbow09 | PS1 | 0,002515718 | 0,002534956 | -0,005821518 | -0,010926338 | 0 | 0 |
| elbow10 | PS14 | -0,009361596 | -0,009487626 | -0,02132529 | -0,028704085 | 0 | 0 |
| elbow10 | PS15B | -0,003761982 | -0,003731003 | -0,016634688 | -0,024609961 | 0 | 0 |
| elbow10 | PS18C | -0,006843811 | -0,006843321 | -0,021126164 | -0,02990647 | 0 | 0 |
| elbow10 | PS19A | 0,001301959 | 0,001299415 | -0,013378498 | -0,022780421 | 0 | 0 |
| elbow10 | PS19F | -0,01377855 | -0,013923292 | -0,024469825 | -0,0309873 | 0 | 0 |
| elbow10 | PS23F | 0,079940205 | 0,081254009 | -0,034504217 | -0,103257152 | 0 | 0 |
| elbow10 | PS3 | -0,008518819 | -0,008590559 | -0,019919451 | -0,02761138 | 0 | 0 |
| elbow10 | PS4 | 0,009427384 | 0,009170085 | -0,028164792 | -0,05215652 | 0 | 0 |
| elbow10 | PS5 | -0,016213641 | -0,016183814 | -0,026693662 | -0,03317421 | 0 | 0 |
| elbow10 | PS6A | -0,000691663 | -0,000751084 | -0,017768321 | -0,028907827 | 0 | 0 |
| elbow10 | PS6B | -0,016213641 | -0,016162776 | -0,02699914 | -0,034136654 | 0 | 0 |
| elbow10 | PS7F | 0,006486719 | 0,006594672 | -0,017339479 | -0,033014461 | 0 | 0 |
| elbow10 | PS9V | -0,009102368 | -0,009109184 | -0,020488525 | -0,02774546 | 0 | 0 |
| elbow10 | PS1 | -0,012670194 | -0,012534106 | -0,024844651 | -0,03242655 | 0 | 0 |
| elbow11 | PS14 | -0,006854511 | -0,006882172 | -0,030890895 | -0,044859074 | 0 | 0 |
| elbow11 | PS18C | -0,018886157 | -0,01865799 | -0,04391423 | -0,060304578 | 0 | 0 |
| elbow11 | PS19A | -0,000847944 | -0,001221645 | -0,02875579 | -0,047132181 | 0 | 0 |
| elbow11 | PS19F | -0,038561046 | -0,038432561 | -0,050796794 | -0,058044868 | 0 | 0 |
| elbow11 | PS23F | -0,023580954 | -0,023644936 | -0,0768755 | -0,107624418 | 0 | 0 |
| elbow11 | PS3 | -0,000817817 | -0,000774354 | -0,022429583 | -0,036705795 | 0 | 0 |
| elbow11 | PS4 | -0,031776547 | -0,031757137 | -0,061983056 | -0,080000097 | 0 | 0 |
| elbow11 | PS5 | 0,045849616 | 0,045582129 | -0,035085422 | -0,086618851 | 0 | 0 |
| elbow11 | PS6A | -0,011758856 | -0,01196638 | -0,036030184 | -0,05219964 | 0 | 0 |
| elbow11 | PS6B | 0,030077354 | 0,030319628 | -0,019880138 | -0,050081974 | 0 | 0 |
| elbow11 | PS7F | -0,007431324 | -0,007548399 | -0,035499019 | -0,053468884 | 0 | 0 |
| elbow11 | PS9V | -0,019907248 | -0,019786164 | -0,039732102 | -0,052895026 | 0 | 0 |
| elbow12 | PS14 | -0,002779618 | -0,002703346 | -0,01150837 | -0,017020662 | 0 | 0 |
| elbow12 | PS15B | -0,003830046 | -0,003820975 | -0,011078451 | -0,015710512 | 0 | 0 |
| elbow12 | PS18C | -0,00261595 | -0,002714293 | -0,011163151 | -0,016530844 | 0 | 0 |
| elbow12 | PS19A | 0,023663295 | 0,023554379 | -0,005199781 | -0,023326121 | 0 | 0 |
| elbow12 | PS23F | -0,014850179 | -0,014876874 | -0,018750452 | -0,021122258 | 0 | 0 |
| elbow12 | PS3 | -0,00593752 | -0,005931101 | -0,011393648 | -0,014791078 | 0 | 0 |
| elbow12 | PS4 | -0,009055514 | -0,009120163 | -0,016469102 | -0,021032805 | 0 | 0 |
| elbow12 | PS5 | -0,011353676 | -0,011380547 | -0,017333012 | -0,020963308 | 0 | 0 |
| elbow12 | PS6A | -0,012652377 | -0,012650104 | -0,017454407 | -0,020131996 | 0 | 0 |
| elbow12 | PS6B | -0,014850179 | -0,014844616 | -0,018840988 | -0,021350063 | 0 | 0 |
| elbow12 | PS7F | -0,00068511 | -0,000627267 | -0,012226886 | -0,019796272 | 0 | 0 |
| elbow12 | PS1 | 0,00068603 | 0,000698561 | -0,014897686 | -0,024532649 | 0 | 0 |
| elbow13 | PS14 | -0,00175778 | -0,001747904 | -0,003321999 | -0,004298834 | 0 | 0 |
| elbow13 | PS15B | -0,00175778 | -0,001768157 | -0,003320728 | -0,004283467 | 0 | 0 |
| elbow13 | PS18C | -0,00175778 | -0,001749278 | -0,003288523 | -0,004344233 | 0 | 0 |
| elbow13 | PS19A | 0,002052512 | 0,002058438 | -0,001398786 | -0,003466896 | 0 | 0 |
| elbow13 | PS19F | 0,002312391 | 0,002309545 | -0,002322506 | -0,00514014 | 0 | 0 |
| elbow13 | PS23F | -0,00175778 | -0,0017425 | -0,003307637 | -0,004236588 | 0 | 0 |
| elbow13 | PS3 | -0,000166268 | -0,000182737 | -0,002503025 | -0,004002567 | 0 | 0 |
| elbow13 | PS4 | -0,00175778 | -0,001748989 | -0,003338993 | -0,004251198 | 0 | 0 |
| elbow13 | PS5 | 0,005235227 | 0,005311777 | -0,00463698 | -0,0107617 | 0 | 0 |
| elbow13 | PS6A | -0,00175778 | -0,001750555 | -0,003278217 | -0,004184296 | 0 | 0 |
| elbow13 | PS6B | -0,00175778 | -0,001762408 | -0,003309494 | -0,004359399 | 0 | 0 |
| elbow13 | PS7F | 0,002088374 | 0,002096209 | -0,003163468 | -0,006523294 | 0 | 0 |
| elbow13 | PS9V | 0,001738723 | 0,001726296 | -0,003126744 | -0,006278752 | 0 | 0 |
| elbow13 | PS1 | -0,000956498 | -0,00094533 | -0,002576545 | -0,003564522 | 0 | 0 |
| elbow14 | PS14 | -0,003288852 | -0,003255458 | -0,010625022 | -0,01513435 | 0 | 0 |
| elbow14 | PS15B | 0,004783396 | 0,004770527 | -0,004129166 | -0,009536757 | 0 | 0 |
| elbow14 | PS18C | -0,001216722 | -0,001271748 | -0,009568571 | -0,014849381 | 0 | 0 |
| elbow14 | PS19A | -0,009687418 | -0,009688945 | -0,014245534 | -0,017012767 | 0 | 0 |
| elbow14 | PS19F | 0,009122758 | 0,008971318 | -0,006043822 | -0,015978295 | 0 | 0 |
| elbow14 | PS23F | 0,035184377 | 0,034992045 | -0,010051369 | -0,038008125 | 0 | 0 |
| elbow14 | PS3 | -0,004251077 | -0,004234026 | -0,009728344 | -0,013573736 | 0 | 0 |
| elbow14 | PS4 | -0,009687418 | -0,00963852 | -0,014354522 | -0,017347563 | 0 | 0 |
| elbow14 | PS5 | -0,009687418 | -0,009630542 | -0,01430219 | -0,017379418 | 0 | 0 |
| elbow14 | PS6A | -0,002843474 | -0,002907863 | -0,012635129 | -0,018902469 | 0 | 0 |
| elbow14 | PS6B | 0,012290604 | 0,012321913 | -0,008860331 | -0,021951512 | 0 | 0 |
| elbow14 | PS7F | -0,004751253 | -0,004753332 | -0,010185935 | -0,013538837 | 0 | 0 |
| elbow14 | PS9V | -0,006849888 | -0,006901111 | -0,012353016 | -0,015840879 | 0 | 0 |
| elbow14 | PS1 | -0,009117617 | -0,009111463 | -0,013938517 | -0,017084962 | 0 | 0 |
| elbow15 | PS14 | -0,003256427 | -0,003251055 | -0,005887417 | -0,007575313 | 0 | 0 |
| elbow15 | PS15B | -0,003256427 | -0,003253842 | -0,005847828 | -0,007356347 | 0 | 0 |

| cluster | type | obs_estimate | unbiased_estimate | ci_limit_0.05 | ci.adj_limit_0.00357142857142857 | signif_ci | signif_ci.adj |
|---|---|---|---|---|---|---|---|
| elbow15 | PS18C | 0,004032655 | 0,004106689 | -0,002471194 | -0,006281463 | 0 | 0 |
| elbow15 | PS19A | -0,003256427 | -0,003256944 | -0,005756347 | -0,007472467 | 0 | 0 |
| elbow15 | PS19F | -0,003256427 | -0,003269656 | -0,005816866 | -0,007350582 | 0 | 0 |
| elbow15 | PS23F | -0,003256427 | -0,003259623 | -0,005784575 | -0,007424552 | 0 | 0 |
| elbow15 | PS3 | -0,003256427 | -0,003247818 | -0,005775373 | -0,007439209 | 0 | 0 |
| elbow15 | PS4 | 0,002238079 | 0,002214969 | -0,006123456 | -0,01116145 | 0 | 0 |
| elbow15 | PS5 | -0,003256427 | -0,003275209 | -0,005814006 | -0,007485228 | 0 | 0 |
| elbow15 | PS6A | -0,003256427 | -0,003239279 | -0,005816625 | -0,007465753 | 0 | 0 |
| elbow15 | PS7F | 4,07774E-05 | 4,69903E-05 | -0,002838266 | -0,0045944 | 0 | 0 |
| elbow15 | PS9V | -0,002898645 | -0,002900932 | -0,005578299 | -0,007255707 | 0 | 0 |
| elbow15 | PS1 | -0,003256427 | -0,003271585 | -0,005790893 | -0,007530236 | 0 | 0 |
| elbow16 | PS14 | -0,00954428 | -0,009369101 | -0,025555227 | -0,035640711 | 0 | 0 |
| elbow16 | PS15B | 0,021009479 | 0,021132416 | -0,015347907 | -0,041005652 | 0 | 0 |
| elbow16 | PS18C | -0,007552277 | -0,007745923 | -0,030323023 | -0,044433098 | 0 | 0 |
| elbow16 | PS19A | -0,003263716 | -0,00315907 | -0,023770259 | -0,037698209 | 0 | 0 |
| elbow16 | PS19F | -0,012712505 | -0,012653929 | -0,034113907 | -0,046991346 | 0 | 0 |
| elbow16 | PS23F | -0,036706175 | -0,03678083 | -0,045628278 | -0,052184823 | 0 | 0 |
| elbow16 | PS3 | 0,016050549 | 0,016214196 | -0,011370389 | -0,028108501 | 0 | 0 |
| elbow16 | PS4 | -0,020084366 | -0,020229649 | -0,036429422 | -0,046545718 | 0 | 0 |
| elbow16 | PS5 | 0,014109676 | 0,013955221 | -0,025747567 | -0,052535307 | 0 | 0 |
| elbow16 | PS6A | -0,008482036 | -0,008400738 | -0,03088539 | -0,046641267 | 0 | 0 |
| elbow16 | PS6B | -0,018543782 | -0,018567232 | -0,038524848 | -0,050840003 | 0 | 0 |
| elbow16 | PS7F | -0,003263435 | -0,003048878 | -0,028135776 | -0,04399703 | 0 | 0 |
| elbow16 | PS9V | 0,024563307 | 0,02438194 | -0,00799473 | -0,029404895 | 0 | 0 |
| elbow17 | PS14 | -0,003023774 | -0,003018643 | -0,006304901 | -0,008578544 | 0 | 0 |
| elbow17 | PS15B | -0,003023774 | -0,00301841 | -0,006230889 | -0,008265925 | 0 | 0 |
| elbow17 | PS18C | -0,001515478 | -0,001504076 | -0,005554026 | -0,008471054 | 0 | 0 |
| elbow17 | PS19A | -0,003023774 | -0,003042784 | -0,006229407 | -0,008141231 | 0 | 0 |
| elbow17 | PS19F | -0,003023774 | -0,003052441 | -0,006246368 | -0,008538263 | 0 | 0 |
| elbow17 | PS23F | -0,003023774 | -0,002968454 | -0,006361797 | -0,008318056 | 0 | 0 |
| elbow17 | PS3 | -0,000660777 | -0,000635547 | -0,004992675 | -0,007560891 | 0 | 0 |
| elbow17 | PS4 | -0,003023774 | -0,003023199 | -0,0062566 | -0,008296199 | 0 | 0 |
| elbow17 | PS5 | -0,003023774 | -0,003018706 | -0,006289608 | -0,008264059 | 0 | 0 |
| elbow17 | PS6A | -0,003023774 | -0,003028651 | -0,006189991 | -0,008362269 | 0 | 0 |
| elbow17 | PS6B | 0,035437765 | 0,035558824 | -0,004012777 | -0,028679834 | 0 | 0 |
| elbow17 | PS7F | -0,003023774 | -0,00304164 | -0,006309884 | -0,008576465 | 0 | 0 |
| elbow17 | PS9V | -0,003023774 | -0,003015057 | -0,006296336 | -0,008319487 | 0 | 0 |
| elbow17 | PS1 | -0,003023774 | -0,003050424 | -0,00621367 | -0,008237676 | 0 | 0 |
| elbow18 | PS14 | -0,002930403 | -0,002952718 | -0,00558016 | -0,00726331 | 0 | 0 |
| elbow18 | PS15B | -0,002930403 | -0,002943756 | -0,005548062 | -0,007215396 | 0 | 0 |
| elbow18 | PS18C | -0,002930403 | -0,002947825 | -0,005553337 | -0,007157587 | 0 | 0 |
| elbow18 | PS19A | -0,002930403 | -0,002914541 | -0,005568667 | -0,00726701 | 0 | 0 |
| elbow18 | PS19F | -0,002930403 | -0,002945216 | -0,005531389 | -0,007088846 | 0 | 0 |
| elbow18 | PS23F | -0,002930403 | -0,002940449 | -0,005549725 | -0,007360436 | 0 | 0 |
| elbow18 | PS3 | -0,002930403 | -0,002936979 | -0,00556406 | -0,007322725 | 0 | 0 |
| elbow18 | PS4 | -0,002930403 | -0,002918885 | -0,00560995 | -0,007184191 | 0 | 0 |
| elbow18 | PS5 | -0,002930403 | -0,002930581 | -0,005585521 | -0,007247068 | 0 | 0 |
| elbow18 | PS6A | -0,002930403 | -0,002936512 | -0,005527392 | -0,007117934 | 0 | 0 |
| elbow18 | PS6B | 0,025274725 | 0,025507119 | -0,003024787 | -0,020293257 | 0 | 0 |
| elbow18 | PS7F | 0,00989011 | 0,009870173 | -0,008592397 | -0,020025634 | 0 | 0 |
| elbow18 | PS9V | -0,002930403 | -0,002929066 | -0,005548601 | -0,00722774 | 0 | 0 |
| elbow18 | PS1 | -0,002930403 | -0,002924189 | -0,005645755 | -0,007335092 | 0 | 0 |
| elbow19 | PS14 | -0,004151702 | -0,004131676 | -0,00736259 | -0,009460495 | 0 | 0 |
| elbow19 | PS15B | -0,004151702 | -0,004173828 | -0,007322739 | -0,009239147 | 0 | 0 |
| elbow19 | PS18C | -0,004151702 | -0,004169313 | -0,007311185 | -0,009236499 | 0 | 0 |
| elbow19 | PS19A | -0,004151702 | -0,00412915 | -0,007295264 | -0,009435459 | 0 | 0 |
| elbow19 | PS19F | 0,006809836 | 0,006793806 | -0,004722778 | -0,012499817 | 0 | 0 |
| elbow19 | PS23F | 0,026068078 | 0,025904628 | -0,004264107 | -0,023501063 | 0 | 0 |
| elbow19 | PS3 | -0,00388645 | -0,003893237 | -0,007199353 | -0,009121959 | 0 | 0 |
| elbow19 | PS4 | -0,0020727 | -0,002005732 | -0,005857186 | -0,008295413 | 0 | 0 |
| elbow19 | PS5 | -0,004151702 | -0,004157902 | -0,007260037 | -0,0092628 | 0 | 0 |
| elbow19 | PS6A | -0,001193122 | -0,001217732 | -0,005534683 | -0,008267942 | 0 | 0 |
| elbow19 | PS6B | 0,005463683 | 0,005392239 | -0,008184779 | -0,015891906 | 0 | 0 |
| elbow19 | PS7F | -0,002127411 | -0,002085489 | -0,006512217 | -0,009217818 | 0 | 0 |
| elbow19 | PS9V | -0,004151702 | -0,004178683 | -0,007286981 | -0,009255462 | 0 | 0 |
| elbow19 | PS1 | -0,004151702 | -0,004135207 | -0,007291948 | -0,009416417 | 0 | 0 |
| elbow20 | PS14 | -0,008026028 | -0,007989885 | -0,012432673 | -0,015000715 | 0 | 0 |
| elbow20 | PS15B | -0,008026028 | -0,008057829 | -0,012291736 | -0,015209653 | 0 | 0 |
| elbow20 | PS18C | -0,008026028 | -0,007994137 | -0,012400241 | -0,015094252 | 0 | 0 |
| elbow20 | PS19A | -0,002108868 | -0,002036176 | -0,011409095 | -0,016889196 | 0 | 0 |
| elbow20 | PS19F | 0,018267678 | 0,0181284 | -0,002389273 | -0,01421565 | 0 | 0 |

| cluster | type | obs_estimate | unbiased_estimate | ci_limit_0.05 | ci.adj_limit_0.00357142857142857 | signif_ci | signif_ci.adj |
|---|---|---|---|---|---|---|---|
| elbow20 | PS23F | -0,008026028 | -0,008027054 | -0,012344557 | -0,014990091 | 0 | 0 |
| elbow20 | PS3 | 0,001588776 | 0,001546856 | -0,008499136 | -0,014693195 | 0 | 0 |
| elbow20 | PS4 | 0,005959986 | 0,005973713 | -0,015047765 | -0,029238029 | 0 | 0 |
| elbow20 | PS5 | 0,011204741 | 0,011123628 | -0,016646692 | -0,0339571 | 0 | 0 |
| elbow20 | PS6A | -0,00033372 | -0,000401969 | -0,012807531 | -0,020324399 | 0 | 0 |
| elbow20 | PS6B | -0,008026028 | -0,008004639 | -0,012345956 | -0,014896915 | 0 | 0 |
| elbow20 | PS7F | -0,008026028 | -0,00802845 | -0,012360752 | -0,015221905 | 0 | 0 |
| elbow20 | PS9V | 0,007358588 | 0,007439117 | -0,015043559 | -0,029304237 | 0 | 0 |
| elbow20 | PS1 | 0,006218986 | 0,006259746 | -0,006810191 | -0,014238123 | 0 | 0 |
| elbow21 | PS14 | -0,013999108 | -0,014025309 | -0,023359877 | -0,0292993 | 0 | 0 |
| elbow21 | PS15B | -0,013999108 | -0,013930761 | -0,023498595 | -0,029734499 | 0 | 0 |
| elbow21 | PS18C | -0,013999108 | -0,013999123 | -0,023412349 | -0,029398662 | 0 | 0 |
| elbow21 | PS19A | -0,005257849 | -0,005293384 | -0,018081632 | -0,026721076 | 0 | 0 |
| elbow21 | PS19F | -0,013999108 | -0,013915179 | -0,023465261 | -0,029713524 | 0 | 0 |
| elbow21 | PS23F | -0,013999108 | -0,014014978 | -0,023335272 | -0,029565806 | 0 | 0 |
| elbow21 | PS3 | -0,013733856 | -0,013690455 | -0,023336512 | -0,029469908 | 0 | 0 |
| elbow21 | PS5 | -0,013999108 | -0,013917095 | -0,023717188 | -0,029693586 | 0 | 0 |
| elbow21 | PS6A | -0,004383723 | -0,004405558 | -0,020230029 | -0,030693921 | 0 | 0 |
| elbow21 | PS6B | -0,013999108 | -0,013982328 | -0,023359162 | -0,029053226 | 0 | 0 |
| elbow21 | PS7F | -0,013999108 | -0,014010714 | -0,023535246 | -0,029590195 | 0 | 0 |
| elbow21 | PS9V | -0,013999108 | -0,01399414 | -0,023294269 | -0,029211048 | 0 | 0 |
| elbow21 | PS1 | -0,013999108 | -0,013980569 | -0,023321075 | -0,029516421 | 0 | 0 |
| elbow22 | PS14 | -0,000111179 | -8,49378E-05 | -0,018401301 | -0,030708997 | 0 | 0 |
| elbow22 | PS15B | 0,00023489 | 0,000116467 | -0,013964764 | -0,022765513 | 0 | 0 |
| elbow22 | PS18C | -0,020137894 | -0,020214731 | -0,031331259 | -0,03895598 | 0 | 0 |
| elbow22 | PS19A | -0,001227934 | -0,001178531 | -0,0172412 | -0,026996362 | 0 | 0 |
| elbow22 | PS19F | 0,003498027 | 0,00329387 | -0,016577539 | -0,027835991 | 0 | 0 |
| elbow22 | PS23F | -0,029779127 | -0,029847923 | -0,037707332 | -0,042468133 | 0 | 0 |
| elbow22 | PS4 | -0,019512797 | -0,019488795 | -0,0313586 | -0,038686309 | 0 | 0 |
| elbow22 | PS5 | -0,000641598 | -0,000441867 | -0,034420612 | -0,05583635 | 0 | 0 |
| elbow22 | PS6A | 0,000198094 | 0,000220451 | -0,019218642 | -0,031952905 | 0 | 0 |
| elbow22 | PS6B | -0,029779127 | -0,02974449 | -0,037842139 | -0,043011834 | 0 | 0 |
| elbow22 | PS7F | -0,006909877 | -0,006826517 | -0,028847614 | -0,043170364 | 0 | 0 |
| elbow22 | PS9V | -0,00339214 | -0,003280348 | -0,016578008 | -0,02520126 | 0 | 0 |
| elbow23 | PS14 | -0,004244454 | -0,004249947 | -0,006683476 | -0,008167148 | 0 | 0 |
| elbow23 | PS15B | -0,004244454 | -0,004257156 | -0,006690627 | -0,008260811 | 0 | 0 |
| elbow23 | PS18C | -0,004244454 | -0,004258881 | -0,006747239 | -0,008294046 | 0 | 0 |
| elbow23 | PS19A | -0,004244454 | -0,004232894 | -0,00668492 | -0,008328068 | 0 | 0 |
| elbow23 | PS19F | -0,004244454 | -0,004220795 | -0,006696249 | -0,008239953 | 0 | 0 |
| elbow23 | PS23F | 0,006744557 | 0,006623276 | -0,00846677 | -0,018622734 | 0 | 0 |
| elbow23 | PS3 | -0,000786979 | -0,000799786 | -0,004434306 | -0,006819665 | 0 | 0 |
| elbow23 | PS4 | -0,004244454 | -0,004233273 | -0,006741563 | -0,008250908 | 0 | 0 |
| elbow23 | PS5 | -0,002412952 | -0,002386303 | -0,006102172 | -0,008554439 | 0 | 0 |
| elbow23 | PS6A | 0,00533571 | 0,005246771 | -0,00485122 | -0,011307488 | 0 | 0 |
| elbow23 | PS6B | 0,014986316 | 0,014867894 | -0,003473834 | -0,014615076 | 0 | 0 |
| elbow23 | PS7F | -0,004244454 | -0,00428102 | -0,006687271 | -0,008253217 | 0 | 0 |
| elbow23 | PS9V | -0,001591934 | -0,001557535 | -0,006277316 | -0,009382479 | 0 | 0 |
| elbow23 | PS1 | 0,007436458 | 0,007506192 | -0,003090762 | -0,010136451 | 0 | 0 |
| elbow24 | PS14 | -0,003864471 | -0,003821904 | -0,018184046 | -0,027842056 | 0 | 0 |
| elbow24 | PS15B | -0,009370744 | -0,009426278 | -0,01761936 | -0,022906963 | 0 | 0 |
| elbow24 | PS18C | -0,003576484 | -0,003723076 | -0,013167943 | -0,019708968 | 0 | 0 |
| elbow24 | PS19A | -0,006416296 | -0,006245617 | -0,017158904 | -0,024554419 | 0 | 0 |
| elbow24 | PS19F | 0,004132041 | 0,00411681 | -0,009269322 | -0,017680425 | 0 | 0 |
| elbow24 | PS23F | -0,005258727 | -0,005289069 | -0,020153488 | -0,029641791 | 0 | 0 |
| elbow24 | PS4 | -0,012584734 | -0,01256084 | -0,018069988 | -0,02132117 | 0 | 0 |
| elbow24 | PS5 | -0,009088231 | -0,009039002 | -0,017750839 | -0,023294639 | 0 | 0 |
| elbow24 | PS6A | -0,00855543 | -0,008444108 | -0,021331236 | -0,029801877 | 0 | 0 |
| elbow24 | PS6B | 0,005119783 | 0,005159047 | -0,016689772 | -0,030376699 | 0 | 0 |
| elbow24 | PS7F | -0,000322686 | -0,000304965 | -0,019146475 | -0,03106339 | 0 | 0 |
| elbow24 | PS9V | -0,008997323 | -0,009013463 | -0,016472192 | -0,021381294 | 0 | 0 |
| elbow25 | PS14 | -0,011356656 | -0,011396531 | -0,01800395 | -0,022333164 | 0 | 0 |
| elbow25 | PS15B | -0,011356656 | -0,011352393 | -0,01809577 | -0,022206034 | 0 | 0 |
| elbow25 | PS18C | -0,007693652 | -0,007689719 | -0,015972279 | -0,021501061 | 0 | 0 |
| elbow25 | PS19A | -0,011356656 | -0,011408787 | -0,017968706 | -0,022164608 | 0 | 0 |
| elbow25 | PS19F | 0,020996076 | 0,02075912 | -0,019839956 | -0,043851625 | 0 | 0 |
| elbow25 | PS23F | 0,027104883 | 0,027902876 | -0,030026592 | -0,068269603 | 0 | 0 |
| elbow25 | PS3 | -0,003630829 | -0,003582568 | -0,011496878 | -0,016206662 | 0 | 0 |
| elbow25 | PS4 | -0,004363649 | -0,004303971 | -0,017160345 | -0,025335443 | 0 | 0 |
| elbow25 | PS5 | -0,011356656 | -0,011399139 | -0,017972269 | -0,021964222 | 0 | 0 |
| elbow25 | PS6A | 0,011720267 | 0,011646645 | -0,022904185 | -0,045295662 | 0 | 0 |
| elbow25 | PS7F | -0,010602508 | -0,01054587 | -0,017430535 | -0,021655276 | 0 | 0 |

| cluster | type | obs_estimate | unbiased_estimate | ci_limit_0.05 | ci.adj_limit_0.00357142857142857 | signif_ci | signif_ci.adj |
|---|---|---|---|---|---|---|---|
| elbow25 | PS9V | -0,011356656 | -0,0112598 | -0,018007638 | -0,022122451 | 0 | 0 |
| elbow25 | PS1 | -0,00622845 | -0,006192538 | -0,014794541 | -0,019755576 | 0 | 0 |
| elbow26 | PS14 | -0,017555353 | -0,017561674 | -0,027892228 | -0,034749956 | 0 | 0 |
| elbow26 | PS15B | -0,017555353 | -0,017599142 | -0,027985998 | -0,034536769 | 0 | 0 |
| elbow26 | PS18C | -0,010549076 | -0,010498776 | -0,023010415 | -0,031152828 | 0 | 0 |
| elbow26 | PS19A | -0,017555353 | -0,01747356 | -0,028075894 | -0,034576961 | 0 | 0 |
| elbow26 | PS19F | -0,0044085 | -0,004381807 | -0,019925434 | -0,029297195 | 0 | 0 |
| elbow26 | PS23F | 0,089587504 | 0,089763536 | -0,02304551 | -0,098094267 | 0 | 0 |
| elbow26 | PS3 | -0,005979484 | -0,00598108 | -0,019915376 | -0,029223116 | 0 | 0 |
| elbow26 | PS4 | 0,009056333 | 0,008927168 | -0,017890095 | -0,033233935 | 0 | 0 |
| elbow26 | PS5 | 0,001675416 | 0,001807383 | -0,027746532 | -0,045231685 | 0 | 0 |
| elbow26 | PS6A | -0,016456452 | -0,01648906 | -0,026940778 | -0,033986834 | 0 | 0 |
| elbow26 | PS6B | 0,007897827 | 0,008095195 | -0,015579241 | -0,029229174 | 0 | 0 |
| elbow26 | PS7F | -0,017555353 | -0,017490931 | -0,028004126 | -0,034277634 | 0 | 0 |
| elbow26 | PS9V | -0,002170737 | -0,002279855 | -0,025950991 | -0,041510098 | 0 | 0 |
| elbow26 | PS1 | 0,001568579 | 0,001623625 | -0,013895239 | -0,02387846 | 0 | 0 |
| elbow27 | PS14 | -0,001502453 | -0,001523818 | -0,004122178 | -0,005703728 | 0 | 0 |
| elbow27 | PS15B | -0,002235053 | -0,002225912 | -0,00365519 | -0,004627072 | 0 | 0 |
| elbow27 | PS18C | -0,000739327 | -0,000731078 | -0,004380691 | -0,006782538 | 0 | 0 |
| elbow27 | PS19A | -0,000826989 | -0,000832811 | -0,003397857 | -0,004980939 | 0 | 0 |
| elbow27 | PS19F | 0,001931613 | 0,001993013 | -0,005531714 | -0,009878158 | 0 | 0 |
| elbow27 | PS23F | -0,002876079 | -0,002857505 | -0,004457334 | -0,005481332 | 0 | 0 |
| elbow27 | PS4 | 0,000786925 | 0,000820425 | -0,004477408 | -0,007629195 | 0 | 0 |
| elbow27 | PS5 | -0,002876079 | -0,002897787 | -0,004439 | -0,005296726 | 0 | 0 |
| elbow27 | PS6A | 0,005376712 | 0,005325048 | -0,005275901 | -0,011972059 | 0 | 0 |
| elbow27 | PS6B | -0,002876079 | -0,002876799 | -0,004446053 | -0,005410001 | 0 | 0 |
| elbow27 | PS7F | -0,001367783 | -0,001376371 | -0,004102353 | -0,005898829 | 0 | 0 |
| elbow27 | PS9V | -0,001337618 | -0,001309567 | -0,004214405 | -0,005967961 | 0 | 0 |
| elbow27 | PS1 | 2,63426E-05 | 2,29367E-05 | -0,002415602 | -0,00399632 | 0 | 0 |
| elbow28 | PS14 | -0,005829096 | -0,005841575 | -0,008751248 | -0,01066672 | 0 | 0 |
| elbow28 | PS15B | -0,005829096 | -0,005829722 | -0,008776441 | -0,010623298 | 0 | 0 |
| elbow28 | PS18C | -0,004627173 | -0,004641966 | -0,008176117 | -0,010691942 | 0 | 0 |
| elbow28 | PS19A | 0,001150961 | 0,001181902 | -0,008509404 | -0,014443404 | 0 | 0 |
| elbow28 | PS19F | -0,001512132 | -0,001530297 | -0,00667525 | -0,00997161 | 0 | 0 |
| elbow28 | PS23F | -0,005829096 | -0,005795647 | -0,008838874 | -0,010800972 | 0 | 0 |
| elbow28 | PS3 | 0,013252048 | 0,013414909 | -0,00413123 | -0,014110131 | 0 | 0 |
| elbow28 | PS4 | -0,005829096 | -0,00581431 | -0,008807298 | -0,010717275 | 0 | 0 |
| elbow28 | PS5 | -0,00033459 | -0,000256716 | -0,008848657 | -0,014085374 | 0 | 0 |
| elbow28 | PS6A | -0,004902312 | -0,004929268 | -0,00803739 | -0,010161346 | 0 | 0 |
| elbow28 | PS7F | -0,005829096 | -0,005819174 | -0,008837363 | -0,010691194 | 0 | 0 |
| elbow28 | PS9V | -0,005267614 | -0,005282679 | -0,008466625 | -0,01052424 | 0 | 0 |
| elbow28 | PS1 | 0,003790478 | 0,003757805 | -0,00929694 | -0,017609339 | 0 | 0 |
| elbow29 | PS14 | -0,002140322 | -0,00215041 | -0,007813572 | -0,01123066 | 0 | 0 |
| elbow29 | PS15B | -0,005217246 | -0,005209361 | -0,008334573 | -0,010244502 | 0 | 0 |
| elbow29 | PS18C | -0,001081596 | -0,001068966 | -0,006677747 | -0,010577125 | 0 | 0 |
| elbow29 | PS19A | -0,005217246 | -0,005199245 | -0,008269259 | -0,010159086 | 0 | 0 |
| elbow29 | PS19F | -0,005217246 | -0,005222195 | -0,008358621 | -0,010183833 | 0 | 0 |
| elbow29 | PS23F | 0,005771765 | 0,005844742 | -0,009853394 | -0,019979354 | 0 | 0 |
| elbow29 | PS3 | 0,003365838 | 0,003363335 | -0,005866486 | -0,011290019 | 0 | 0 |
| elbow29 | PS4 | 0,005771765 | 0,005779433 | -0,010484348 | -0,020313164 | 0 | 0 |
| elbow29 | PS5 | 0,002475062 | 0,002498551 | -0,009241623 | -0,016393405 | 0 | 0 |
| elbow29 | PS6A | -0,001554242 | -0,001487983 | -0,00773962 | -0,011793001 | 0 | 0 |
| elbow29 | PS6B | 0,012534234 | 0,012459982 | -0,013051803 | -0,029388595 | 0 | 0 |
| elbow29 | PS7F | -0,005217246 | -0,005217543 | -0,008337411 | -0,010353744 | 0 | 0 |
| elbow29 | PS9V | -0,005217246 | -0,005198385 | -0,008376136 | -0,01025649 | 0 | 0 |
| elbow29 | PS1 | 0,000943723 | 0,001017409 | -0,00501616 | -0,008426932 | 0 | 0 |
| elbow30 | PS14 | -0,007158681 | -0,007215755 | -0,016187593 | -0,021938447 | 0 | 0 |
| elbow30 | PS15B | -0,007158681 | -0,007214552 | -0,016220406 | -0,021588648 | 0 | 0 |
| elbow30 | PS18C | -0,001358925 | -0,001453977 | -0,011798569 | -0,018298165 | 0 | 0 |
| elbow30 | PS19A | -0,000748424 | -0,000735241 | -0,013419275 | -0,021142848 | 0 | 0 |
| elbow30 | PS19F | -0,007158681 | -0,007108113 | -0,016339974 | -0,021587322 | 0 | 0 |
| elbow30 | PS23F | -0,007158681 | -0,007117271 | -0,016243456 | -0,021702009 | 0 | 0 |
| elbow30 | PS3 | -0,006439773 | -0,006427827 | -0,015515565 | -0,021092336 | 0 | 0 |
| elbow30 | PS4 | -0,007158681 | -0,007174904 | -0,016277312 | -0,021854384 | 0 | 0 |
| elbow30 | PS5 | -0,007158681 | -0,007367158 | -0,016135111 | -0,021278066 | 0 | 0 |
| elbow30 | PS6A | 0,069764396 | 0,071248767 | -0,042847861 | -0,118875207 | 0 | 0 |
| elbow30 | PS6B | 0,002456704 | 0,002553438 | -0,014200821 | -0,02421697 | 0 | 0 |
| elbow30 | PS7F | -0,006404533 | -0,006437813 | -0,015478995 | -0,021321937 | 0 | 0 |
| elbow30 | PS9V | -0,007158681 | -0,007089532 | -0,016387496 | -0,022551909 | 0 | 0 |
| elbow30 | PS1 | -0,007158681 | -0,007124359 | -0,016111016 | -0,022046668 | 0 | 0 |
| elbow31 | PS14 | -0,010730079 | -0,010668044 | -0,019620407 | -0,025330726 | 0 | 0 |

| cluster | type | obs_estimate | unbiased_estimate | ci_limit_0.05 | ci.adj_limit_0.00357142857142857 | signif_ci | signif_ci.adj |
|---|---|---|---|---|---|---|---|
| elbow31 | PS15B | -0,014778662 | -0,014810105 | -0,021113671 | -0,025266382 | 0 | 0 |
| elbow31 | PS18C | -0,006462763 | -0,00650506 | -0,016517703 | -0,023100924 | 0 | 0 |
| elbow31 | PS19A | -0,014778662 | -0,01477139 | -0,021078242 | -0,025169041 | 0 | 0 |
| elbow31 | PS19F | -0,006876564 | -0,00698061 | -0,017923216 | -0,024623922 | 0 | 0 |
| elbow31 | PS23F | 0,042913645 | 0,043111065 | -0,018471824 | -0,058837117 | 0 | 0 |
| elbow31 | PS4 | -0,007785655 | -0,007962299 | -0,018892927 | -0,025900175 | 0 | 0 |
| elbow31 | PS5 | -0,007785655 | -0,007769632 | -0,019384727 | -0,025835026 | 0 | 0 |
| elbow31 | PS6A | -0,001958149 | -0,001915812 | -0,021230405 | -0,033000574 | 0 | 0 |
| elbow31 | PS6B | 0,011742893 | 0,011697777 | -0,010060187 | -0,024487232 | 0 | 0 |
| elbow31 | PS7F | -0,014778662 | -0,014772149 | -0,021270608 | -0,025128847 | 0 | 0 |
| elbow31 | PS9V | 0,004102457 | 0,004013069 | -0,018828588 | -0,034066119 | 0 | 0 |
| elbow31 | PS1 | 0,008952762 | 0,008988231 | -0,008469589 | -0,019775613 | 0 | 0 |
| elbow32 | PS14 | -0,004009024 | -0,004006343 | -0,006990242 | -0,008852615 | 0 | 0 |
| elbow32 | PS15B | -0,004009024 | -0,004034052 | -0,006855584 | -0,008659844 | 0 | 0 |
| elbow32 | PS18C | 0,02355165 | 0,023456418 | -0,003337296 | -0,018773504 | 0 | 0 |
| elbow32 | PS19A | -0,004009024 | -0,004014748 | -0,006960654 | -0,008767757 | 0 | 0 |
| elbow32 | PS19F | -0,004009024 | -0,004012184 | -0,007019053 | -0,008830223 | 0 | 0 |
| elbow32 | PS23F | -0,004009024 | -0,004017442 | -0,006938623 | -0,008692345 | 0 | 0 |
| elbow32 | PS3 | -0,002473271 | -0,002444306 | -0,005868497 | -0,007870452 | 0 | 0 |
| elbow32 | PS4 | -0,004009024 | -0,004017451 | -0,00698024 | -0,008885097 | 0 | 0 |
| elbow32 | PS5 | 0,003683283 | 0,003752899 | -0,007664058 | -0,014624295 | 0 | 0 |
| elbow32 | PS6A | -0,004009024 | -0,004005558 | -0,006950535 | -0,008729498 | 0 | 0 |
| elbow32 | PS6B | -0,004009024 | -0,004017925 | -0,006960639 | -0,008869954 | 0 | 0 |
| elbow32 | PS7F | -0,004009024 | -0,003995287 | -0,006969606 | -0,008788513 | 0 | 0 |
| elbow32 | PS9V | -0,004009024 | -0,004003561 | -0,006958245 | -0,008664635 | 0 | 0 |
| elbow32 | PS1 | 0,015328582 | 0,015319149 | -0,000558486 | -0,011090123 | 0 | 0 |
| elbow33 | PS14 | -0,004458527 | -0,004446506 | -0,00764191 | -0,009696976 | 0 | 0 |
| elbow33 | PS15B | -0,004458527 | -0,004460602 | -0,00764936 | -0,00962458 | 0 | 0 |
| elbow33 | PS18C | 0,009116133 | 0,009149549 | -0,010759172 | -0,02365769 | 0 | 0 |
| elbow33 | PS19A | -0,004458527 | -0,004452224 | -0,007634145 | -0,009593916 | 0 | 0 |
| elbow33 | PS19F | -0,001381604 | -0,001346135 | -0,006433156 | -0,009507685 | 0 | 0 |
| elbow33 | PS23F | -0,004458527 | -0,004474801 | -0,007642066 | -0,009546894 | 0 | 0 |
| elbow33 | PS3 | 0,003218731 | 0,003168378 | -0,005349912 | -0,010478812 | 0 | 0 |
| elbow33 | PS4 | -0,004458527 | -0,004464052 | -0,00757389 | -0,009663072 | 0 | 0 |
| elbow33 | PS5 | -0,002627026 | -0,002675878 | -0,006350963 | -0,008705816 | 0 | 0 |
| elbow33 | PS6A | -0,004458527 | -0,00450159 | -0,007539713 | -0,009421045 | 0 | 0 |
| elbow33 | PS7F | -0,004458527 | -0,004447223 | -0,007549511 | -0,009518906 | 0 | 0 |
| elbow33 | PS9V | -0,004458527 | -0,004456104 | -0,007663481 | -0,009562799 | 0 | 0 |
| elbow33 | PS1 | 0,001951729 | 0,001916146 | -0,004809794 | -0,009100864 | 0 | 0 |
| elbow34 | PS14 | -0,006176446 | -0,006204288 | -0,012639172 | -0,017008793 | 0 | 0 |
| elbow34 | PS15B | -0,009253369 | -0,009231446 | -0,013684282 | -0,016396267 | 0 | 0 |
| elbow34 | PS18C | 0,001880448 | 0,001900289 | -0,006162249 | -0,01157847 | 0 | 0 |
| elbow34 | PS19A | -0,009253369 | -0,009273382 | -0,013661125 | -0,016507389 | 0 | 0 |
| elbow34 | PS19F | 0,016106356 | 0,016015546 | -0,005678195 | -0,019226875 | 0 | 0 |
| elbow34 | PS23F | -0,007116617 | -0,007152037 | -0,012861974 | -0,016384336 | 0 | 0 |
| elbow34 | PS3 | -0,008837569 | -0,008795815 | -0,013326855 | -0,016274729 | 0 | 0 |
| elbow34 | PS4 | -0,002260362 | -0,002169504 | -0,013753094 | -0,020913496 | 0 | 0 |
| elbow34 | PS5 | 0,008728649 | 0,009037606 | -0,010934784 | -0,022627755 | 0 | 0 |
| elbow34 | PS6A | -0,009253369 | -0,00930375 | -0,013654433 | -0,016366949 | 0 | 0 |
| elbow34 | PS6B | 0,042761283 | 0,042654552 | -0,000318245 | -0,027679148 | 0 | 0 |
| elbow34 | PS7F | -0,009253369 | -0,00925582 | -0,013683489 | -0,016644716 | 0 | 0 |
| elbow34 | PS9V | -0,009253369 | -0,009223693 | -0,013841916 | -0,016461245 | 0 | 0 |
| elbow34 | PS1 | 0,001181104 | 0,001170596 | -0,006907386 | -0,011511939 | 0 | 0 |
| elbow35 | PS14 | -0,015730517 | -0,015664799 | -0,024808738 | -0,030422804 | 0 | 0 |
| elbow35 | PS15B | -0,012086792 | -0,012212054 | -0,026441219 | -0,03606272 | 0 | 0 |
| elbow35 | PS19A | 0,005861926 | 0,006098181 | -0,022515663 | -0,039951599 | 0 | 0 |
| elbow35 | PS19F | -0,012786093 | -0,012831423 | -0,024358727 | -0,03203218 | 0 | 0 |
| elbow35 | PS23F | 0,018682439 | 0,019001778 | -0,019205151 | -0,04231492 | 0 | 0 |
| elbow35 | PS3 | 0,011119407 | 0,01125888 | -0,009715274 | -0,022951481 | 0 | 0 |
| elbow35 | PS4 | -0,0197791 | -0,019663918 | -0,027550622 | -0,032055763 | 0 | 0 |
| elbow35 | PS5 | -0,0197791 | -0,019745503 | -0,027393235 | -0,032096029 | 0 | 0 |
| elbow35 | PS6A | -0,014971407 | -0,014950319 | -0,024526881 | -0,030378158 | 0 | 0 |
| elbow35 | PS7F | -0,0197791 | -0,019775448 | -0,027570229 | -0,032223643 | 0 | 0 |
| elbow35 | PS9V | 0,002392846 | 0,002356738 | -0,021315838 | -0,036024219 | 0 | 0 |
| elbow35 | PS1 | 0,013678878 | 0,013735151 | -0,012583486 | -0,029294312 | 0 | 0 |

| cluster | type | obs_estimate | unbiased_estimate | ci_limit_0.05 | ci.adj_limit_0.00357142857142857 | signif_ci | signif_ci.adj |
|---|---|---|---|---|---|---|---|

**Supplementary data 2. Statistical results from linear regression model bootstrapping for Spn cross-sectional phenotype and serotype analysis.** The observed (obs_estimate) and estimated (unbiased_estimate) proportions of clusters among total B cells for a given serotype-specificity and sample, were generated with an upper-sided linear regression model with wild bootstrap simulation (9999 resamples). Confidence intervals (ci) and Bonferroni's method adjusted ci (ci.adj) are shown, as well as statistical significance (signif_ci/ signif_ci.adj = 1).
