# Supplementary material for: Combinatorial multimer staining and spectral flow cytometry facilitate quantification and characterization of polysaccharide-specific B cell immunity
Source: Commun Biol. 2023 Oct 28;6:1095. doi: 10.1038/s42003-023-05444-3 (PMC10613281; doi:10.1038/s42003-023-05444-3)
Supplement: Supplementary file 6 — Reporting Summary [file 42003_2023_5444_MOESM6_ESM.pdf]

nature portfolio

Corresponding author(s): Dennis Hoving
Simon P. Jochems

Last updated by author(s): Aug 9, 2023

Reporting Summary

Nature Portfolio wishes to improve the reproducibility of the work that we publish. This form provides structure for consistency and transparency in reporting. For further information on Nature Portfolio policies, see our Editorial Policies and the Editorial Policy Checklist.

Statistics

For all statistical analyses, confirm that the following items are present in the figure legend, table legend, main text, or Methods section.

n/a	Confirmed	
☐	☒	The exact sample size (*n*) for each experimental group/condition, given as a discrete number and unit of measurement
☐	☒	A statement on whether measurements were taken from distinct samples or whether the same sample was measured repeatedly
☐	☒	The statistical test(s) used AND whether they are one- or two-sided<br>*Only common tests should be described solely by name; describe more complex techniques in the Methods section.*
☐	☒	A description of all covariates tested
☐	☒	A description of any assumptions or corrections, such as tests of normality and adjustment for multiple comparisons
☐	☒	A full description of the statistical parameters including central tendency (e.g. means) or other basic estimates (e.g. regression coefficient) AND variation (e.g. standard deviation) or associated estimates of uncertainty (e.g. confidence intervals)
☐	☒	For null hypothesis testing, the test statistic (e.g. *F*, *t*, *r*) with confidence intervals, effect sizes, degrees of freedom and *P* value noted<br>*Give P values as exact values whenever suitable.*
☒	☐	For Bayesian analysis, information on the choice of priors and Markov chain Monte Carlo settings
☒	☐	For hierarchical and complex designs, identification of the appropriate level for tests and full reporting of outcomes
☐	☒	Estimates of effect sizes (e.g. Cohen's *d*, Pearson's *r*), indicating how they were calculated

Our web collection on statistics for biologists contains articles on many of the points above.

Software and code

Policy information about availability of computer code

Data collection	Cytek Spectraflow (cytometry data) and SkanIt Software 3.1.0. (ELISA's)
Data analysis	RStudio version 2021.09.1, OMIQ and GraphPad Prism version 9.3.1

For manuscripts utilizing custom algorithms or software that are central to the research but not yet described in published literature, software must be made available to editors and reviewers. We strongly encourage code deposition in a community repository (e.g. GitHub). See the Nature Portfolio guidelines for submitting code & software for further information.

Data

Policy information about availability of data

All manuscripts must include a data availability statement. This statement should provide the following information, where applicable:

- Accession codes, unique identifiers, or web links for publicly available datasets
- A description of any restrictions on data availability
- For clinical datasets or third party data, please ensure that the statement adheres to our policy

Raw and processed data used to generate the figures in this manuscript can be made available upon request.

Research involving human participants, their data, or biological material

Policy information about studies with human participants or human data. See also policy information about sex, gender (identity/presentation), and sexual orientation and race, ethnicity and racism.

Reporting on sex and gender	PBMCs from PCV13-vaccinated group included female and male adults. The South African donors from the GBS study were all pregnant females. PBMC samples from healthy non-PCV13 vaccinated and Dutch individuals were donated anonymously and obtained from Sanquin bloodbank, and therefore no information on sex or gender is available.
Reporting on race, ethnicity, or other socially relevant groupings	The South African donors from the GBS study were primarily Black South African. PBMC samples from PCV13-vaccinated individuals were primarily White European. Non-PCV13 vaccinated and Dutch individuals were donated anonymously and obtained from Sanquin bloodbank, and therefore no information on race or ethnicity is available, however given the population, these are likely predominantly White European.
Population characteristics	See above
Recruitment	The South African donors from the GBS study were invited to participate in the study upon visiting prenatal clinic. Other participants were targeted via study promotional material or bloodbank reach out.
Ethics oversight	PBMCs from PCV13-vaccinated adults were collected from the tissue bank at the Liverpool School of Tropical Medicine (HTA license: 12548). The GBS study with South African donors was approved by the Human Research Ethics Committee (Medical) of the University of the Witwatersrand (M090937). Blood from healthy donors with unknown vaccination status (likely non-PCV13) were collected concentrated peripheral blood (Buffy coat) that was donated anonymously by healthy adult volunteers at Sanquin bloodbank (Amsterdam, The Netherlands). Informed written consent was obtained from all participating donors

Note that full information on the approval of the study protocol must also be provided in the manuscript.

Field-specific reporting

Please select the one below that is the best fit for your research. If you are not sure, read the appropriate sections before making your selection.

☒ Life sciences ☐ Behavioural & social sciences ☐ Ecological, evolutionary & environmental sciences

For a reference copy of the document with all sections, see nature.com/documents/nr-reporting-summary-flat.pdf

Life sciences study design

All studies must disclose on these points even when the disclosure is negative.

Sample size	For the Streptococcus pneumoniae study, one longitudinal donor and was included and for the cross-sectional analysis 5 unvaccinated and 4 PCV13-vaccinated individuals. For the GBS project, 8 Dutch donors and 22 South African donors were included.
Data exclusions	For the GBS study, 11 donors were excluded (leaving 22 for analysis) as less then 100.000 B cells were recorded. Furthermore, from both studies, cell populations from donors with less then 5 PS-specific cells were excluded from analysis.
Replication	Technical duplicates and experimental repeats were recored and shown in the manuscript.
Randomization	Samples and groups were randomised on day of analysis, to minimise batch-associated bias.
Blinding	The investigators were not blinded to group allocation during analysis.

Reporting for specific materials, systems and methods

We require information from authors about some types of materials, experimental systems and methods used in many studies. Here, indicate whether each material, system or method listed is relevant to your study. If you are not sure if a list item applies to your research, read the appropriate section before selecting a response.

Materials & experimental systems

n/a	Involved in the study
☐	☒ Antibodies
☒	☐ Eukaryotic cell lines
☒	☐ Palaeontology and archaeology
☒	☐ Animals and other organisms
☒	☐ Clinical data
☒	☐ Dual use research of concern
☒	☐ Plants

Methods

n/a	Involved in the study
☒	☐ ChIP-seq
☐	☒ Flow cytometry
☒	☐ MRI-based neuroimaging

Antibodies

Antibodies used

For the S. pneumoniae study:
CD3 BV510 OKT3 Mouse lgG2a. κ Biolegend 317332
CD56 BV510 HCD56 Mouse lgGl. κ Biolegend 318340
CD19 BUV395 HIB19 Mouse lgGl. κ BD 740287
HLA-DR BUV496 G46-6 Mouse lgG2a. κ BD 749866
lgD BUV563 I-A6-2 Mouse lgG2a. κ BD 741394
lgM BV570 MHM-88 Mouse lgGl. κ Biolegend 314517
lgA APC-Vio7701S11-8E10 Mouse lgGlκ Miltenyi 130-113-999
lgG PE-CF594 G18-145 Mouse lgGl. κ BD 562538
CD138 BUV737 Ml15 Mouse lgGl. κ BD 612834
CD38 APC-Fire810 HIT2 Mouse lgGl. κ Biolegend 303550
CD1O BV605 HI 10 a Mouse lgGl. κ Biolegend 312222
CD11cc BV650 Bu15 Mouse lgGl. κ Biolegend 337237
CD20 Pacific orange Hl47 Mouse lgG3 ThermoFisher MHCD2030
CD21 BUV805 BLy4 Mouse lgGl. κ BD 742008
CD27 APC-R700 M-T271 Mouse lgGl. κ BD 565116
CD43 PerCP-CyS.5 lG 10 Mouse lgG1. κ BD 563521
CD45RBMEM55 PE MEM-55 Mouse lgG2b. κ Biolegend 310204
CD5 BV750 L17F12 Mouse lgG2a. κ BD 747090
CD73 AF647 AD2 Mouse lgGl Abeam 243083
CD95 PE-Cy5 DX2 Mouse lgGl. κ Biolegend 305610
CD80PerCP-eFluor71016-lOAl Hamster lgG ThermoFisher 46-0801-82
CXCR3 PE-Cy7 G025H7 Mouse lgGl. κ Biolegend 353719
PD-1 BV480 EH12.1 Mouse lgGl. κ BD 566112
IRF4 APC REA201 Human lgGl Miltenyi 130-100-915
Caspase-3 V450 C92-605 Rabbit lgG BD 560627
SA-BB515 B8515 BD 564453
SA-BUV615 BUV615 BD 613013
SA-BUV661 BUV661 BD 612979
SA-BV421 BV421 BD 563259
SA-BV711 BV711 Biolegend 405241
SA-BV785 BV785 Biolegend 405249
Live/Dead Blue ThermoFisher L34962

For the GBS study:
CD7 BV510 M-T701 Mouse lgGl. κ BD 563650
CD19 BUV395 HIB19 Mouse lgGl. κ BD 740287
HLA-DR BUV496 G46-6 Mouse lgG2a. κ BD 749866
lgD BUV563 I-A6-2 Mouse lgG2a. κ BD 741394
lgM BV570 MHM-88 Mouse lgGl. κ Biolegend 314517
lgA APC-Vio7701S11-8E10 Mouse lgGlκ Miltenyi 130-113-999
lgG PE-CF594 G18-145 Mouse lgGl. κ BD 562538
CD38 APC-Fire810 HIT2 Mouse lgGl. κ Biolegend 303550
CD1O BV605 HI 10 a Mouse lgGl. κ Biolegend 312222
CD11c BV650 Bu15 Mouse lgGl. κ Biolegend 337237
CD20 Pacific orange Hl47 Mouse lgG3 ThermoFisher MHCD2030
CD21 BUV805 BLy4 Mouse lgGl. κ BD 742008
CD27 APC-R700 M-T271 Mouse lgGl. κ BD 565116
CD43 PerCP-CyS.5 lG 10 Mouse lgG1. κ BD 563521
CD45RBMEM55 PE MEM-55 Mouse lgG2b. κ Biolegend 310204
CD5 BV750 L17F12 Mouse lgG2a. κ BD 747090
CD73 AF647 AD2 Mouse lgGl Abeam 243083
CD95 PE-Cy5 DX2 Mouse lgGl. κ Biolegend 305610
CD80PerCP-eFluor71016-lOAl Hamster lgG ThermoFisher 46-0801-82
CXCR3 PE-Cy7 G025H7 Mouse lgGl. κ Biolegend 353719
PD-1 BV480 EH12.1 Mouse lgGl. κ BD 566112
LAIRl BUV737 DX26 (RUO) Mouse lgGl. κ BD 749446
IRF4 APC REA201 Human lgGl Miltenyi 130-100-915

Ki-67 BV711 Ki67 Mouse lgGl. κ iolegend 350515
T-bet BV785 4B10 Mouse lgGl. κ iolegend 644835
SA-BB515 BB515 BD 564453
SA-BUV615 BUV615 BD 613013
SA-BUV661 BUV661 BD 612979
SA-BV421 BV421 BD 563259
Live/Dead Blue ThermoFisher L34962

Validation

Antibodies were validated by suppliers, in other publications (among others, Glass et al. Immunity 2020) and by ourselves. We established optimal dilutions by titration of each antibody individually and validation in full panel.

Flow Cytometry

Plots

Confirm that:

☒ The axis labels state the marker and fluorochrome used (e.g. CD4-FITC).

☒ The axis scales are clearly visible. Include numbers along axes only for bottom left plot of group (a 'group' is an analysis of identical markers).

☒ All plots are contour plots with outliers or pseudocolor plots.

☒ A numerical value for number of cells or percentage (with statistics) is provided.

Methodology

Sample preparation

Five to ten million cryopreserved PBMCs in 10% Dimethyl sulfoxide (DMSO), 20% foetal calf serum (FCS) in Roswell Park Memorial Institute (RPMI) 1640 were thawed by dropwise addition of 5ml RPMI with 20% FCS prewarmed to 37oC, and washed once more with the same medium. For Spn, the centrifugation steps after each wash were performed for 5 minutes at 4oC and 450xg (live cells) and 600xg (after fixation). Because we had lower cell numbers in the South African samples, the centrifugation for fixed cells was increased to 800xg for 7 minutes at 4oC to reduce sample loss. For GBS samples, red blood cells were removed by incubating the PBMC with 5ml RBC lysis buffer (Apotheek AZL, 97930725) for 5 minutes at room temperature, and washed first with RPMI and then FACS buffer (2mM EDTA and 0.5% BSA in PBS). FACS buffer was used for all subsequent wash steps, unless indicated otherwise.

Cells were stained for viability and blocked for CD16/32 with anti-human FC Receptor binding inhibitor (ThermoFisher Scientific, 14-9161-73) in PBS for 15 minutes at room temperature (Spn) or 20 minutes on ice (GBS). Then, after one wash, the cells were first stained for extracellular surface markers with BD Brilliant stain buffer plus (BD, 566385) in FACS buffer for 15 minutes at room temperature (Spn) or 20 minutes on ice (GBS) and washed. For the GBS staining, cells were then blocked with lOµg/ml anti-Siglec 9 (RnD systems, MAB1139-100) in FACS buffer to reduce unspecific binding of sialic acids on the PS. A sample fraction from all donors was combined up to 400,000 cells total for an FMM (fluorescence minus multimer) control that were stained for all markers, except PS-SA multimers. The remaining cells were stained with 5µg/ml (Spn) or 2.Sµg/ml (GBS) PS-SA multimers with BD stain plus in FACS buffer for 30 minutes on ice and washed. Multimers were centrifuged prior to use at 2500xg for 5minutes at 4oC to pull down aggregates that may cause artifacts in the staining. Then a fixation/ permeabilization (eBioscience, 00-5523-00) step was performed for 30 minutes at room temperature. Finally, cells were washed twice in permeabilization buffer (eBioscience, 00-5523-00) and stained for intracellular markers with lX BD stain plus in permeabilization buffer for 30 minutes on ice. Cells were then washed in permeabilization buffer, and once more in FACS buffer. Stained cells resuspended in FACS and acquired on the same day of preparation.

Instrument

5 laser Cytek Aurora spectral flow cytometer

Software

RStudio version 2021.09.1, Graphpad Prism version 9.3.1 and OMIQ

Cell population abundance

The samples were sorted but analysed via spectral flow cytometry.

Gating strategy

Serotype-specific B cells (live singlet SSClowCD3-CD56-CD19+HLADR+PS-SA+ (Spn) or SSClowCD7-CD19+HLADR+PS-SA+ (GBS) were gated and exported per PS-specific gate. High dimensional analysis and clustering was performed based on differential expression of 25 additional markers.

☒ Tick this box to confirm that a figure exemplifying the gating strategy is provided in the Supplementary Information.
